# Pharmaceutical Prescription in Canine Acute Diarrhoea: A Longitudinal Electronic Health Record Analysis of First Opinion Veterinary Practices

**DOI:** 10.3389/fvets.2019.00218

**Published:** 2019-07-02

**Authors:** David A. Singleton, P. J. M. Noble, Fernando Sánchez-Vizcaíno, Susan Dawson, Gina L. Pinchbeck, Nicola J. Williams, Alan D. Radford, Philip H. Jones

**Affiliations:** ^1^Epidemiology and Population Health, Institute of Infection and Global Health, University of Liverpool, Neston, United Kingdom; ^2^Institute of Veterinary Science, University of Liverpool, Neston, United Kingdom; ^3^Bristol Veterinary School, University of Bristol, Bristol, United Kingdom

**Keywords:** health informatics, antimicrobial resistance, companion animal, electronic health record, pharmacosurveillance, acute canine diarrhoea, haemorrhagic diarrhoea

## Abstract

Canine acute diarrhoea is frequently observed in first opinion practice, though little is known about commonly used diagnostic or therapeutic management plans, including use of antimicrobials. This retrospective observational study utilised electronic health records augmented with practitioner-completed questionnaires from 3,189 cases (3,159 dogs) collected from 179 volunteer veterinary practices between April 2014 and January 2017. We used multivariable analysis to explore factors potentially associated with pharmaceutical agent prescription, and resolution of clinical signs by 10 days post-initial presentation. Use of bacteriological and/or parasitological diagnostic tests were uncommon (3.2% of cases, 95% confidence interval, CI, 2.4–4.0), though systemic antimicrobials were the most commonly prescribed pharmaceutical agents (49.7% of cases, 95% CI 46.1–53.2). Such prescription was associated with haemorrhagic diarrhoea (odds ratio, OR, 4.1; 95% CI 3.4–5.0), body temperature in excess of 39.0°C, or moderate/severe cases (OR 1.3, 95% CI 1.1–1.7). Gastrointestinal agents (e.g., antacids) were prescribed to 37.7% of cases (95% CI 35.4–39.9), and were most frequently prescribed to vomiting dogs regardless of presence (OR 46.4, 95% CI 19.4–110.8) or absence of blood (OR 17.1, 95% CI 13.4–21.9). Endoparasiticides/endectocides were prescribed to 7.8% of cases (95% CI 6.8–9.0), such prescription being less frequent for moderate/severe cases (OR 0.5, 95% CI 0.4–0.7), though more frequent when weight loss was recorded (OR 3.4, 95% CI 1.3–9.0). Gastrointestinal nutraceuticals (e.g., probiotics) were dispensed to 60.8% of cases (95% CI 57.1–64.6), these cases less frequently presenting with moderate/severe clinical signs (OR 0.6, 95% CI 0.5–0.8). Nearly a quarter of cases were judged lost to follow-up (*n*=754). Insured (OR 0.7, 95% CI 0.5–0.9); neutered (OR 0.4, 95% CI 0.3–0.5), or vaccinated dogs (OR 0.3, 95% CI 0.3–0.4) were less commonly lost to follow-up. Of remaining dogs, clinical signs were deemed resolved in 95.4% of cases (95% CI 94.6–96.2). Provision of dietary modification advice and gastrointestinal nutraceuticals alone were positively associated with resolution (OR 2.8, 95% CI 1.3–6.1); no such associations were found for pharmaceutical agents, including antimicrobials. Hence, this study supports the view that antimicrobials are largely unnecessary for acute diarrhoea cases; this being of particular importance when considering the global threat posed by antimicrobial resistance.

## Introduction

Acute diarrhoea commonly affects dogs (1). Whilst the majority of cases are generally mild and self-limiting, some can be life threatening (2–7). Aetiology is complex, including a range of non-infectious lifestyle factors, such as a history of scavenging or being fed home-cooked diets (1, 5, 6). Zoonotic (e.g., *Campylobacter, Salmonella, Giardia* spp.) and non-zoonotic (e.g., canine parvovirus, canine enteric coronavirus) pathogens have also been implicated (6, 8–14), though the precise role some of these play remains of debate (8, 15–18). A range of therapeutic options are available, either targeting potential infectious agents and/or clinical signs (2, 5). Together this creates a complex clinical decision-making environment for practitioners when first presented with such cases, further compounded by relatively infrequent use of diagnostic testing (5).

Antimicrobial prescription, as a management strategy, is a particular focus for research due to the increasing threat posed by antimicrobial resistance (19). Antimicrobial prescription has been recorded in between 45 and 70% of canine diarrhoea cases (2, 5, 7, 20, 21), with prescription being most frequent in cases presenting with pyrexia or haemorrhagic diarrhoea (2, 5). These findings most likely reflect a perception that such clinical signs increase likelihood of infectious process involvement and/or intestinal mucosal compromise, increasing risk of bacteraemia (15). However, recent case-control studies of canine acute haemorrhagic diarrhoea syndrome (AHDS) have questioned whether antimicrobial therapy has an impact on odds of recovery in non-septic patients (10, 22), and indeed whether antimicrobials should be prescribed at all (10, 16, 23).

In addition to antimicrobial prescription, management strategies frequently encompass other pharmaceutical agents both as primary diarrhoea therapies (24), or to manage associated clinical signs (2). The potential utility of gastrointestinal nutraceuticals (including prebiotics, probiotics, adsorbents, and motility modifiers) has also attracted recent attention (25, 26), though evidence of *in vivo* efficacy remains limited (25).

The complex and often undetermined aetiology of acute canine diarrhoea, as well as the range of therapeutic or management interventions available, provides a natural opportunity to more fully understand factors that might drive complex clinical decision-making in practice, as well as which of these decisions might impact outcome. This study aimed to combine electronic health record (EHR) and questionnaire data collected from a large network of UK veterinary practices to explore factors associated with the decision to prescribe pharmaceutical agents or dispense nutraceuticals to dogs presenting with acute diarrhoea.

## Materials and Methods

### Data Collection

This longitudinal retrospective study analysed electronic health records (EHRs) collected from 179 volunteer veterinary practices (347 sites) situated in the United Kingdom (UK) that participate in the Small Animal Veterinary Surveillance Network (SAVSNET) and utilise Robovet practice management software (Vet Solutions Ltd.). A veterinary practice was defined as a single business, whereas “sites” included all branches that comprised an individual veterinary practice. SAVSNET hold ethical approval from the University of Liverpool (RETH000964); data collection protocols are more fully described elsewhere (4). Briefly, EHRs were collected from consultations where a booked appointment was made to see a veterinary professional (veterinary surgeon or veterinary nurse) between 1 April 2014 and 31 January 2017. Every consultation was classified by the consulting veterinary professional into one of ten categories indicating the main reason that the animal presented and the main presenting complaint (MPC) (21). In addition to the MPC, a further questionnaire was completed in a random selection of consultations by the attending veterinary professional (Table 1). Consultations which had been classified into the “gastroenteric” MPC, which also had an associated completed questionnaire attached were selected for inclusion in this study.

**Table 1:** Questions provided to consulting veterinary professionals in ~10% of consultations (selected at random) where they had selected “gastrointestinal” as the main reason the owner presented the animal to the practice.

**Question**	**Answer options**
1. Please indicate the clinical signs present	Diarrhoea without blood
	Diarrhoea with blood
	Vomiting without blood
	Vomiting with blood
	Melaena
	Weight loss/failure to gain weight
	Poor appetite
	Other
2. If diarrhoea was present how would you describe it?	Small intestinal diarrhoea
	Large intestinal diarrhoea/colitis
	Mixed pattern
	No diarrhoea
	Don't know
3. Please indicate disease severity	Mild illness i.e., normal apart from GI disease
	Moderately ill
	Severely ill/debilitated
4. How does this consultation relate to this episode of illness?	First presentation
	Revisit/check-up
	Don't know
5. How long approximately has the pet had this episode of illness?	Up to 2 days
	Between 3 days and 2 weeks
	More than 2 weeks—less than 1 month
	1 month and over
	Don't know
6. What diagnostic options will be used today for this episode of illness?	None
	Faecal parasitology/bacteriology
	Faecal virology
	Virus serology
	Diagnostic imaging
	Haematology/biochemistry
	Serum B12/Folate and/or serum TLI
	Canine/feline specific pancreatic lipase
	Urinalysis
	Other
7. What advice did you give today?	Change of diet
	Fasting
	Admit patient for treatment
	Refer patient
	Check-up in near future
	Other

A case was defined as a dog presenting for investigation of acute diarrhoea (Table 1, question 1) of 2 days or less duration (Table 1, question 5), where the relevant consultation was the first time the animal had presented for investigation of that diarrhoeic episode (Table 1, question 4). Consultations were selected for presence of diarrhoea but not at the exclusion of other clinical signs. In addition to a range of signalment data (e.g., age, sex, breed etc.), the MPC, and the associated questionnaire responses, each EHR also included a text-based product description and free text clinical narrative. The latter was manually interrogated to summarise animal body temperature (if recorded). Each EHR also contained a vaccination history; animals were defined as currently vaccinated if they had received a vaccination of any composition within 3.5 years preceding the relevant consultation date (27).

### Pharmaceutical, Nutraceutical, or Veterinary Professional Advice Identification

Pharmaceutical agent prescriptions were identified and classified via the semi-structured text-based product description field of the EHR (28). Antimicrobials and anti-inflammatories were further classified by authorised administration route as systemic (oral or injectable forms, hence “systemic”) or topical administration (aural, ocular, skin). Five pharmaceutical families commonly prescribed for management of canine gastroenteric disease (5) were selected for further analyses: systemic antimicrobials (excluding topical antimicrobials), systemic anti-inflammatories (excluding topical anti-inflammatories), gastrointestinal agents e.g., antacids, gastro-protectants, anti-emetics etc., endoparasiticides and endectocides, and products used for euthanasia (henceforth, “euthanasia”). Additionally, the product description field was interrogated to identify dispensed gastrointestinal nutraceutical products. These were defined as products not listed as either authorised veterinary or human medicinal products which contained a range of probiotics, prebiotics, kaolin etc., and were marketed for the purpose of aiding diarrhoea resolution.

### Case Follow-Up

As the majority of canine self-limiting diarrhoea cases resolve within a week (29), cases were considered as resolved if they did not return to the veterinary practice for a mainly gastroenteric reason (as judged by MPC) between 11 and 30 days post-initial presentation. The clinical narratives for all cases re-presenting for examination between 1 and 10 days post-initial presentation were additionally read to record explicit mention of diarrhoeic clinical sign resolution, and any further pharmaceutical prescriptions provided in this time period. Cases were defined as lost to follow-up if they did not re-present to the veterinary practice at all by 31 January 2018, or were seen again within 10 days post-initial presentation but did not re-present by 31 January 2018.

### Statistical Analyses

All analyses were carried out using R language (version 3.4.4). Descriptive proportions and associated 95% confidence intervals (95% CI) were calculated to adjust for clustering (bootstrap method, *n* = 5,000 samples) within site,^1^ encompassing a range of binary or categorical demographic, clinical sign, pharmaceutical agent prescription, and clinical outcome variables. Median and range were calculated for continuous variables. Following descriptive analyses, univariable and multivariable mixed effects logistic regression were used to model a range of outcomes, using the R package “lme4”^2^ Primary outcomes, modelled as binary variables, included resolution of diarrhoea clinical signs (as defined above), and loss to follow-up. The decision to prescribe systemic antimicrobials, systemic anti-inflammatories, gastrointestinal agents, endoparasiticides and endectocides, and to euthanise the dog at initial presentation were also explored, using prescription of such agents as binary independent variables in separate models. A likelihood ratio test (LRT) of a null model indicated that observations were clustered within practice and site; hence both were included as random effects in all models. Univariable regression was first performed, with explanatory variables being retained if a LRT indicated *P* ≤ 0.20 against a null model.

In total, 21 binary or categorical explanatory variables were considered. For all models, these included factors related to animal signalment (insurance status, vaccination status, sex, neutered status, microchip status); questionnaire responses (presence of haemorrhagic diarrhoea, melaena, vomiting, decreased appetite, weight loss, diarrhoeic pattern, clinical severity) and categorised body temperature as recorded within the clinical narrative attached to each consultation (interpreted normal or below 39.0°C, 39.0–39.4°C, 39.5–39.9°C, in excess of 40.0°C, and temperature not recorded), as recorded within the clinical narrative attached to each consultation. Considering clinical severity, due to a low number of severe cases such cases were combined into a single category with moderate cases for all models. Animal's age at consultation was fitted as a continuous explanatory variable; where relevant, polynomial terms were fitted if an LRT indicated significantly improved fit.

For models considering resolution or loss to follow-up only, questionnaire responses indicating that the consulting veterinary professional had provided advice to either modify the animal's diet or fast the animal were also considered. Prescription of pharmaceutical agents or dispensed gastrointestinal nutraceuticals were also included in resolution and loss to follow-up models as binary dependent variables.

In order to take into account the therapeutic complexities surrounding case management, we took three separate approaches to modelling the association of each of the five therapeutic options (e.g., the four investigated pharmaceutical families and gastrointestinal nutraceuticals) and dietary modification advice against case resolution. Firstly, we considered each option/advice regardless of presence of other options e.g., a single case must be prescribed a systemic antimicrobial, but could also be prescribed/dispensed/advised any other option. Secondly, we considered each option/advice in isolation e.g., a single case could only be prescribed a systemic antimicrobial, with no further prescription/dispensing/advice provided. Finally, we also considered dietary advice in combination with each option in isolation e.g., a single case must be provided with dietary modification advice and prescribed a systemic antimicrobial, with no further options provided.

Multivariable analyses underwent step-wise backward elimination to reduce Akaike Information Criterion (AIC) and Bayesian Information Criterion (BIC). Two-way interaction terms were assessed for improved multivariable model fit via a combination of AIC and BIC. Multicollinearity was assessed in the final model via use of the Variance Inflation Factor (VIF), available through the R package “car”^3^ Odds ratios, confidence intervals, correlation of fixed effects and projected probabilities were calculated utilising the R package “sjPlot”^4^ Statistical significance was defined throughout as *P* < 0.05.

## Results

### Study Population

**Figure 1:**
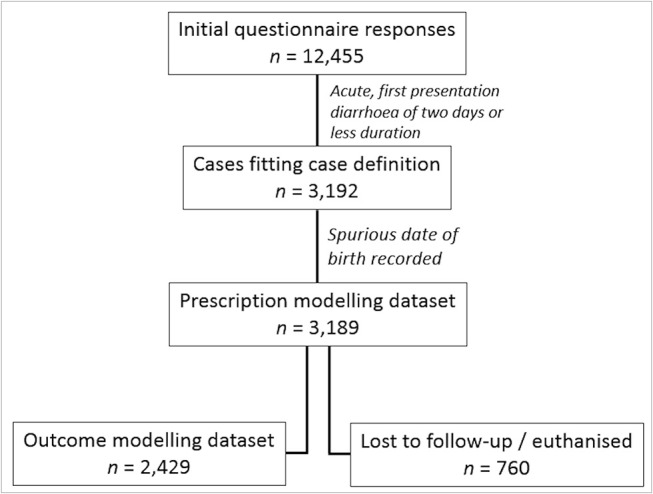
Flow diagram showing case selection procedure for prescription modelling, loss to follow-up, and outcome modelling.

In total, 12,455 questionnaires were completed for canine patients (11,589 unique dogs) with a gastroenteric MPC, of which 3,192 questionnaires (3,162 unique dogs) fitted the acute diarrhoea case definition (two days or less duration and first presentation for examination). Three dogs were removed where a spurious date of birth was recorded (e.g., 1st January 1900). Hence, 3,189 diarrhoea cases (involving 3,159 unique dogs) collected from 179 veterinary practices (347 sites) were included in analyses (Figure 1). Of these retained cases, 50.2% (95% CI, 48.3–52.1) were recorded as male; 62.2% of male cases were neutered (95% CI 59.6–64.9), 72.4% of female cases were neutered (95% CI 69.7–75.1), 54.6% of total retained cases were microchipped (95% CI 52.1–57.2), 24.4% of total retained cases were insured (95% CI 22.1–26.7), and 73.6% of total retained cases has been vaccinated within the preceding 3.5 years (95% CI 71.5–75.8). Median age at initial presentation was 4.2 years (range 0.0–18.5).

### Descriptive Analyses

Cases were considered by clinical severity (Table 1, question 3), clinical sign combinations (Table 1, question 1), and body temperature as recorded in the clinical narrative. The majority of dogs [*n* = 1,893; 59.4% of cases (95% CI 57.8–61.0)] initially presented with non-haemorrhagic diarrhoea (Table 2). Most cases were recorded as mild (*n* = 2,665; 83.6% of cases, 95% CI 82.2–85.0), with moderate cases more commonly reporting diarrhoea with blood, vomiting, weight loss, poor appetite, and a mixed diarrhoeic pattern compared to mild cases. Utilisation of diagnostic tests was uncommon (<10% of all cases), with bacteriology and parasitology being the most commonly performed test (3.2% of cases, 95% CI 2.4–4.0). Dietary modification was the most commonly provided advice to dog owners. In total, 1,812 cases explicitly recorded body temperature within the clinical narrative, reporting a median body temperature of 38.6°C (range 36.2–41.3); a further 53 and 3 cases recorded a “normal” or “increased” temperature, respectively without stating a value. Considered together, an interpreted normal or below 39.0°C body temperature was recorded in 58.4% of cases (*n* = 1,865, 95% CI 56.5–60.4); 39.0°C−39.4°C in 13.1% (*n* = 418, 95% CI 11.8–14.4), 39.5–39.9°C in 3.5% (*n* = 110, 95% CI 2.8–4.1), and >40.0°C in 0.9% of cases (*n* = 30, 95% CI 0.6–1.3). Temperature was not recorded or interpreted in 23.9% of cases (*n* = 763, 95% CI 21.9–25.9). A greater proportion of cases were classified as moderate or severe as reported temperature increased (data not presented).

**Table 2:** Descriptive summary of questionnaire responses for both the entire study population and when stratified by the consulting veterinary professional's assessed case severity, according to questionnaire responses.

**Question**	**Response**	**All cases** **(*n* = 3,189 cases)**	**Mild case** **(*n* = 2,665 cases)**	**Moderate case** **(*n* = 507 cases)**	**Severe case** **(*n* = 17 cases)**
		**% (95% CI)^**a**^**	**% (95% CI)**	**% (95% CI)**	**% (95% CI)**
1. Clinical signs	Diarrhoea without blood	59.4 (57.8–61.0)	60.2 (58.4–62.0)	55.9 (52.0–59.7)	41.8 (17.4–66.1)
	Diarrhoea with blood	40.6 (39.0–42.3)	39.9 (38.1–41.6)	44.2 (40.4–48.0)	58.5 (34.3–82.7)
	Vomit without blood	33.7 (31.8–35.6)	28.9 (27.0–30.8)	58.8 (54.0–63.6)	41.4 (16.7–66.0)
	Vomit with blood	2.3 (1.7–2.9)	2.0 (1.4–2.5)	3.8 (2.1–5.4)	11.8 (0.0–27.3)
	Melaena	0.4 (0.2–0.6)	0.2 (0.0–0.3)	1.6 (0.5–2.7)	5.9 (0.0–17.0)
	Weight loss^b^	1.2 (0.8–1.6)	0.8 (0.5–1.1)	3.2 (1.4–4.9)	6.0 (0.0–17.2)
	Poor appetite	13.8 (12.4–15.2)	10.3 (8.9–11.6)	31.5 (27.3–35.8)	35.4 (11.9–58.8)
	Other	1.6 (1.0–2.2)	1.2 (0.7–1.7)	3.4 (1.5–5.2)	17.9 (0.0–36.4)
2. Pattern	Small intestinal	32.7 (30.5–34.8)	31.8 (29.5–34.1)	37.5 (32.7–42.4)	29.6 (7.5–51.8)
	Large intestinal	39.0 (36.9–41.1)	41.3 (39.2–43.5)	27.0 (22.6–31.4)	35.4 (12.0–58.8)
	Mixed pattern	19.7 (18.0–21.4)	18.2 (16.6–19.9)	26.8 (22.3–31.2)	22.8 (0.0–46.1)
	Don't know	8.7 (7.4–9.9)	8.6 (7.3–10.0)	8.7 (6.3–11.1)	11.9 (0.0–27.1)
3. Diagnostic options	Total	9.0 (7.7–10.3)	7.7 (6.4–9.0)	13.7 (10.2–17.1)	70.3 (47.9–92.6)
	Bacteriology/parasitology	3.2 (2.4–4.0)	3.2 (2.4–4.1)	2.8 (1.3–4.3)	6.0 (0.0–17.3)
	Faecal virology	0.1 (0.0–0.3)	0.0 (0.0–0.1)	0.4 (0.0–0.9)	5.9 (0.0–17.1)
	Virus serology	0.1 (0.0–0.1)	0.0 (0.0–0.1)	0.2 (0.0–0.6)	0.0 (0.0–0.0)
	Diagnostic imaging	0.9 (0.5–1.3)	0.6 (0.3–1.0)	2.0 (0.5–3.5)	11.7 (0.0–25.8)
	Haematology/biochemistry	2.4 (1.8–3.1)	1.4 (0.9–1.8)	6.1 (3.8–8.4)	52.7 (27.9–77.5)
	Serum B12 and/or TLI	0.1 (0.0–0.2)	0.1 (0.0–0.2)	0.2 (0.0–0.6)	0.0 (0.0–0.0)
	Specific pancreatic lipase	0.6 (0.3–0.9)	0.4 (0.2–0.6)	1.6 (0.4–2.8)	11.8 (0.0–27.4)
	Urinalysis	0.3 (0.1–0.4)	0.3 (0.1–0.5)	0.2 (0.0–0.6)	0.0 (0.0–0.0)
	Other	3.1 (2.4–3.9)	2.9 (2.1–3.6)	4.1 (2.3–5.9)	17.6 (0.0–35.8)
4. Advice	Change of diet	69.8 (67.3–72.4)	71.1 (68.5–73.8)	65.0 (60.2–69.8)	5.6 (0.0–15.5)
	Fasting	19.1 (16.4–21.8)	18.4 (15.5–21.3)	23.0 (18.4–27.5)	5.9 (0.0–17.2)
	Admission	2.3 (1.7–3.0)	1.2 (0.7–1.6)	6.5 (4.2–8.8)	58.7 (34.7–82.7)
	Refer	0.2 (0.0–0.3)	0.1 (0.0–0.2)	0.2 (0.0–0.6)	5.6 (0.0–15.8)
	Check-up	22.9 (20.9–24.9)	20.1 (18.0–22.2)	37.5 (32.9–42.0)	17.0 (0.0–38.8)
	Other	49.4 (46.6–52.2)	50.3 (47.2–53.3)	45.3 (40.9–49.8)	35.8 (12.5–59.1)

a *Percentage of cases (95% confidence interval)*.

b *Weight loss or failure to gain weight*.

Pharmaceutical prescription occurred in 78.4% (95% CI 76.3–80.5) of initial presentations, rising to 81.3% of cases (95% CI 79.5–83.2) within 10 days post-initial presentation (Table 3). Systemic antimicrobials were the most commonly prescribed pharmaceutical agent (49.7% of cases at initial presentation, rising to 52.5% within 10 days of initial presentation). Gastrointestinal nutraceuticals were also frequently dispensed (60.8% of cases at initial presentation, rising to 61.7% within 10 days of initial presentation). In total, 4.3% of cases (95% CI 3.4–5.2) had no record of a pharmaceutical agent being prescribed or a gastrointestinal nutraceutical dispensed. Metronidazole represented the most commonly prescribed systemic antimicrobial (47.0% of antimicrobial prescribing cases, 95% CI 41.0–53.1); glucocorticoids the most commonly prescribed systemic anti-inflammatory (81.3% of anti-inflammatory prescribing cases, 95% CI 73.6–89.1); maropitant the most commonly prescribed gastrointestinal agent (44.6% of gastrointestinal prescribing cases, 95% CI 39.9–49.3), and a combination of milbemycins and quinolines were the most commonly prescribed endoparasiticides/endectocides (48.0% of endoparasiticide/ endectocide prescribing cases, 95% CI 41.5–54.5) (see Supplementary Table 1).

**Table 3:** Descriptive summary of pharmaceutical prescriptions and dispensing of nutraceutical products both at initial presentation and when the subsequent 9 days (inclusive) post-presentation were considered.

**Category**	**All cases**	**Mild case**	**Moderate case**	**Severe case**
	**% (95% CI)^**a**^**	**% (95% CI)**	**% (95% CI)**	**% (95% CI)**
**THERAPY—INITIAL PRESENTATION**				
Pharmaceutical agent	78.4 (76.3–80.5)	76.4 (74.0–78.9)	89.4 (86.8–92.0)	58.9 (36.7–81.1)
Systemic antimicrobial	49.7 (46.1–53.2)	48.2 (44.5–51.9)	58.5 (53.4–63.7)	5.9 (0.0–17.2)
Systemic anti-inflammatory	14.2 (10.6–17.8)	14.2 (10.5–17.9)	15.0 (10.1–19.9)	0.0 (0.0–0.0)
Gastrointestinal agent	37.7 (35.4–39.9)	33.3 (31.0–35.6)	60.9 (56.5–65.4)	17.5 (0.3–34.6)
Endoparasiticide and/or endectocide	7.8 (6.8–9.0)	8.7 (7.5–10.0)	3.6 (2.0–5.2)	0.0 (0.0–0.0)
Gastrointestinal nutraceutical	60.8 (57.1–64.6)	63.0 (59.1–66.9)	51.1 (46.0–56.3)	11.9 (0.0–27.5)
Euthanasia/death	0.2 (0.0–0.3)	0.0 (0.0–0.0)	0.0 (0.0–0.0)	35.3 (11.9–58.8)
**THERAPY—INITIAL PRESENTATION AND/OR WITHIN 10 DAYS OF INITIAL PRESENTATION**				
Pharmaceutical agent	81.3 (79.5–83.2)	79.6 (77.4–81.8)	91.1 (88.7–93.6)	59.0 (37.0–81.1)
Systemic antimicrobial	52.5 (49.1–55.8)	51.2 (47.7–54.6)	61.1 (56.2–66.1)	5.9 (0.0–17.3)
Systemic anti-inflammatory	15.3 (11.6–19.0)	15.4 (11.8–19.1)	15.3 (10.5–20.2)	0.0 (0.0–0.0)
Gastrointestinal agent	39.0 (36.7–41.2)	34.7 (32.3–37.2)	62.0 (57.6–66.4)	17.6 (0.4–34.8)
Endoparasiticide and/or endectocide	9.5 (8.3–10.7)	10.6 (9.2–11.9)	4.3 (2.4–6.1)	0.0 (0.0–0.0)
Gastrointestinal nutraceutical	61.7 (58.1–65.4)	63.7 (59.8–67.5)	53.4 (47.9–58.9)	12.0 (0.0–27.5)
Euthanasia/death	0.4 (0.2–0.6)	0.2 (0.0–0.3)	0.2 (0.0–0.6)	35.4 (12.1–58.8)
**OUTCOME**				
Resolution (10 day)	72.6 (70.3–75.0)	73.9 (71.4–76.3)	67.7 (63.2–72.0)	29.5 (9.1–49.9)
Lost to follow-up	23.7 (21.4–26.0)	22.5 (20.2–24.9)	29.0 (24.7–33.2)	35.3 (13.8–56.8)

*Longitudinal outcome is also displayed, with all comparisons shown when considered by consulting veterinary professional assessment of case severity at initial presentation*.

a *Percentage of cases (95% confidence interval)*.

Pharmaceutical prescription frequency varied by case severity, with systemic antimicrobials largely being prescribed to mild and moderate cases (Table 3), and showed considerable variation between cases, particularly in relation to co-prescription (Table 4). Systemic antimicrobial prescription was more frequent in cases reporting diarrhoea with blood compared to diarrhoea without blood, regardless of presence or absence of vomiting (see Supplementary Table 2).

**Table 4:** Descriptive summary of cases where multiple sets of advice, nutraceutical dispensing, or pharmaceutical prescriptions were provided, expressed as a percentage of total cases where each “event type” was provided.

**Event type**		**Percentage (%) of total advice/dispensing/prescription events, by event type**						
	**Total events**	**Diet change**	**Fast**	**Gastrointestinal nutraceutical**	**Systemic antimicrobial**	**Systemic anti-inflammatory**	**Gastrointestinal agent**	**Endoparasiticide/endectocide**
Diet change	2,227		14.0	64.3	49.0	13.4	37.0	7.7
Fast	608	52.0		54.1	50.0	22.2	43.8	5.1
Gastrointestinal nutraceutical	1,939	73.9	17.0		40.7	8.9	33.5	9.0
Systemic antimicrobial	1,585	68.9	19.0	49.8		18.2	37.2	6.0
Systemic anti-inflammatory	454	65.6	30.0	38.1	63.7		26.7	4.0
Gastrointestinal agent	1,200	68.6	22.0	54.2	49.2	10.1		4.2
Endoparasiticide/ endectocide	250	68.4	12.0	70.0	38.0	7.2	20.0	

*For example, a gastrointestinal nutraceutical was also dispensed to 64.3% of cases where “diet change” advice was provided (n = 2,227)*.

### Factors Associated With Pharmaceutical or Nutraceutical Intervention

No variables were significant on univariable analyses for systemic anti-inflammatory prescription (see Supplementary Table 3); hence no further statistical analysis was performed for this prescription category.

#### Systemic Antimicrobial Prescription

**Figure 2:**
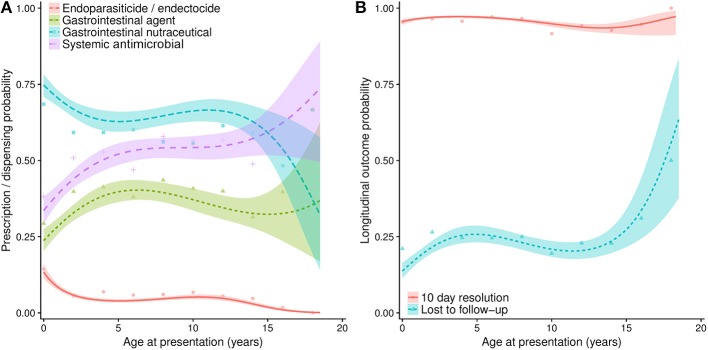
**(A)** Projections from a series of multivariable logistic regression models, estimating the probability of a range of pharmaceutical agents being prescribed at initial presentation for diarrhoea, when considered against age at presentation (in years). **(B)** Estimates of longitudinal outcome, including probability of a case being lost to follow-up or being considered resolved of diarrhoeic clinical signs 10 days post-initial presentation. Lines refer to predicted probability, with shading relating to 95% confidence intervals to such predictions. Points are plotted to show original data points expressing the percentage of animals of each relevant age group (rounded to 2 year groups) that were prescribed a pharmaceutical agent, or were classified into the resolved or lost to follow-up categories.

Dogs presenting with diarrhoea with blood were more frequently prescribed a systemic antimicrobial (Odds ratio, OR, 4.1, 95% CI, 3.4–5.0), compared to diarrhoea without blood; moderate or severe cases were also more frequently prescribed a systemic antimicrobial compared to mild cases (OR 1.3, 95% CI 1.1–1.7) (Table 5). Compared to an interpreted normal or below 39°C body temperature at initial presentation, all other temperature categories were more frequently associated with prescription, peaking at between 39.5 and 39.9°C (OR 5.9, 95% CI 3.6–9.9). Prescription probability increased with age up to ~7 years of age, but remained static between seven and thirteen, and increased from thirteen years of age upwards (Figure 2A). Results from univariable analyses are available in Supplementary Table 4. A cubic polynomial term was included to model age at consultation; no interaction terms significantly improved the fit of the model.

**Table 5:** Parameter estimates from a finalised mixed effects logistic regression model, modelling on a case-level the presence of systemic antimicrobial and gastrointestinal agent prescription against a range of risk factors.

**Random effect**	**Variance**	**SD^**a**^**	**Variable**	**Category**	**β**	**SE^**b**^**	**OR^**c**^ (95% CI)^**d**^**	***P***
**SYSTEMIC ANTIMICROBIAL PRESCRIPTION**								
Practice	0.75	0.87		Intercept	−0.72	0.14	0.49 (0.37–0.64)	
Site	0.27	0.52	Diarrhoea	Without blood	-	-	1.00	-
				**With blood**	**1.42**	**0.10**	**4.13 (3.42–4.98)**	**<0.01**
			Weight loss	Absent	-	-	1.00	-
				Present	0.71	0.40	2.03 (0.93–4.45)	0.08
			Severity	Mild	-	-	1.00	-
				**Moderate/severe**	**0.29**	**0.12**	**1.34 (1.06–1.69)**	**0.01**
			Diarrhoeic pattern	Large intestinal	-	-	1.00	-
				Mixed	0.01	0.13	1.01 (0.78–1.30)	0.95
				Small intestinal	0.07	0.11	1.07 (0.87–1.31)	0.54
				**Unknown**	**−0.53**	**0.17**	**0.59 (0.42–0.82)**	**<0.01**
			Body temperature	Normal/ <39°C	-	-	1.00	-
				Not recorded	0.18	0.11	1.19 (0.97–1.46)	0.09
				**39.0°C** **≤** **39.4°C**	**0.72**	**0.13**	**2.05 (1.58–2.65)**	**<0.01**
				**39.5°C** **≤** **39.9°C**	**1.78**	**0.26**	**5.93 (3.56–9.88)**	**<0.01**
				**40.0°C** **≤**	**1.50**	**0.48**	**4.47 (1.76–11.36)**	**<0.01**
			Age (years)	**Age—linear**	**0.22**	**0.08**	**1.25 (1.07–1.46)**	**0.01**
				**Age—quadratic**	**−0.25**	**0.08**	**0.78 (0.67–0.91)**	**<0.01**
				**Age—cubic**	**0.10**	**0.05**	**1.10 (1.01–1.20)**	**0.04**
**GASTROINTESTINAL AGENT PRESCRIPTION**								
Practice	0.32	0.56		Intercept	−1.65	0.13	0.19 (0.15–0.25)	
Site	0.28	0.53	Vomit	No vomit	-	-	1.00	-
				**Without blood**	**2.84**	**0.13**	**17.13 (13.41–21.89)**	**<0.01**
				**With blood**	**3.84**	**0.45**	**46.35 (19.39–110.81)**	**<0.01**
			Poor appetite	Absent	-	-	1.00	-
				**Present**	**0.65**	**0.15**	**1.92 (1.45–2.55)**	**<0.01**
			Severity	Mild	-	-	1.00	-
				**Moderate & severe**	**0.93**	**0.19**	**2.52 (1.76–3.62)**	**<0.01**
			Diarrhoeic pattern	Large intestinal	-	-	1.00	-
				Mixed	0.28	0.14	1.33 (1.00–1.76)	0.05
				Small intestinal	0.20	0.11	1.22 (0.97–1.53)	0.08
				Unknown	0.01	0.18	1.01 (0.70–1.44)	0.98
			Body temperature	Normal/ <39°C	-	-	1.00	-
				**Not recorded**	**−0.38**	**0.12**	**0.68 (0.54–0.87)**	**<0.01**
				39.0°C ≤ 39.4°C	−0.04	0.15	0.96 (0.72–1.28)	0.78
				39.5°C ≤ 39.9°C	−0.17	0.25	0.84 (0.51–1.39)	0.50
				**40.0°C** **≤**	**−1.05**	**0.50**	**0.35 (0.13–0.93)**	**0.04**
			Vomit & Severity	**No blood: moderate/severe**	**−0.71**	**0.25**	**0.49 (0.30–0.80)**	**0.01**
				**With blood: moderate/severe**	**-2.07**	**0.73**	**0.13 (0.03–0.53)**	**0.01**
			Age (years)	Age—linear	0.13	0.09	1.13 (0.95–1.35)	0.17
				**Age—quadratic**	**−0.33**	**0.09**	**0.72 (0.60–0.87)**	**<0.01**
				Age—cubic	0.09	0.05	1.10 (0.99–1.21)	0.08

*Bold values are indicate significant findings*.

a *Standard Deviation*.

b *Standard Error*.

c *Odds Ratio*.

d *95% Confidence Interval*.

#### Gastrointestinal Agent Prescription

Compared to non-vomiting dogs, dogs vomiting with or without blood were much more frequently prescribed a gastrointestinal agent (Table 5). Non-vomiting moderate and severe cases were also more frequently prescribed compared to non-vomiting mild cases (OR 2.5, 95% CI 1.8–3.6). Prescription probability increased up to approximately 6 years of age, before decreasing until 15 years of age (Figure 2A). Univariable results are available in Supplementary Table 5. A cubic polynomial term was included to model age at consultation; an interaction term between case severity and vomiting significantly improved the fit of the model.

#### Endoparasiticide/Endectocide Prescription

Animals reported to have lost weight were associated with increased odds (OR 3.4, 95% CI 1.3–9.0) of endoparasiticide and/or endectocide prescription, though moderate and severe cases (OR 0.4, 95% CI 0.3–0.7) or vomiting cases without blood (OR 0.5, 95% CI 0.4–0.7) were less frequently prescribed (Table 6). Vaccinated animals were also less frequently (OR 0.6, 95% CI 0.4–0.7) prescribed at initial presentation. Prescription probability decreased sharply up to 3 years of age, remained broadly stable until 12 years of age, and then decreased further (Figure 2A). Univariable results are available in Supplementary Table 6. A cubic polynomial term was included to model age at consultation; no interaction terms significantly improved the fit of the model.

**Table 6:** Parameter estimates from a finalised mixed effects logistic regression model, modelling on a case-level the presence of endoparasiticide/endectocide prescription and dispensing of gastrointestinal nutraceuticals against a range of risk factors.

**Random effect**	**Variance**	**SD^**a**^**	**Variable**	**Category**	**β**	**SE^**b**^**	**OR^**c**^ (95% CI)^**d**^**	***P***
**ENDOPARASITICIDE/ENDECTOCIDE PRESCRIPTION**								
Practice	0.28	0.53		Intercept	−2.38	0.19	0.09 (0.06–0.14)	
Site	0.21	0.46	Vomit	No vomit	-	-	1.0	-
				**Without blood**	**−0.69**	**0.18**	**0.50 (0.36–0.71)**	**<0.01**
				With blood	−1.04	0.74	0.35 (0.08–1.50)	0.16
			Weight loss	Absent	-	-	1.0	-
				**Present**	**1.22**	**0.50**	**3.37 (1.27–9.00)**	**0.02**
			Severity	Mild	-	-	1.0	-
				**Moderate & severe**	**−0.83**	**0.27**	**0.44 (0.26–0.74)**	**<0.01**
			Vaccination status	Unvaccinated	-	-	1.0	-
				**Vaccinated**	**−0.59**	**0.15**	**0.55 (0.42–0.74)**	**<0.01**
			Age (years)	Age—linear	0.07	0.18	1.07 (0.76–1.51)	0.70
				**Age—quadratic**	**0.57**	**0.14**	**1.77 (1.35–2.32)**	**<0.01**
				**Age—cubic**	**−0.36**	**0.11**	**0.70 (0.57–0.87)**	**<0.01**
**GASTROINTESTINAL NUTRACEUTICAL DISPENSING**								
Practice	0.64	0.80		Intercept	0.93	0.16	2.52 (1.85–3.43)	
Site	0.21	0.45	Diarrhoea	Without blood	-	-	1.0	-
				**With blood**	**−0.31**	**0.09**	**0.74 (0.62–0.88)**	**<0.01**
			Vomit	No vomit	-	-	1.0	-
				**Without blood**	**−0.57**	**0.09**	**0.57 (0.47–0.68)**	**<0.01**
				**With blood**	**−1.34**	**0.29**	**0.26 (0.15–0.46)**	**<0.01**
			Other signs	Absent	-	-	1.0	-
				**Present**	**−0.99**	**0.33**	**0.37 (0.19–0.71)**	**<0.01**
			Severity	Mild	-	-	1.0	-
				**Moderate & severe**	**−0.48**	**0.12**	**0.62 (0.49–0.78)**	**<0.01**
			Diarrhoeic pattern	Large intestinal	-	-	1.0	-
				**Mixed**	**0.29**	**0.13**	**1.33 (1.04–1.71)**	**0.02**
				Small intestinal	0.21	0.11	1.23 (1.00–1.51)	0.05
				Unknown	−0.20	0.16	0.82 (0.60–1.12)	0.22
			Body temperature	Normal/ <39°C	-	-	1.0	-
				**Not recorded**	**−0.50**	**0.10**	**0.61 (0.50–0.74)**	**<0.01**
				39.0°C ≤ 39.4°C	−0.15	0.13	0.86 (0.67–1.11)	0.25
				**39.5°C** **≤** **39.9°C**	**−0.66**	**0.22**	**0.52 (0.34–0.80)**	**<0.01**
				40.0°C ≤	−0.57	0.42	0.57 (0.25–1.28)	0.17
			Vaccination status	Unvaccinated	-	-	1.0	-
				Vaccinated	0.15	0.10	1.16 (0.96–1.40)	0.13
			Age (years)	Age—linear	0.03	0.08	1.03 (0.89–1.20)	0.70
				**Age—quadratic**	**0.26**	**0.08**	**1.29 (1.11–1.51)**	**<0.01**
				**Age—cubic**	**−0.14**	**0.05**	**0.87 (0.80–0.95)**	**<0.01**

*Bold values are indicate significant findings*.

a *Standard Deviation*.

b *Standard Error*.

c *Odds Ratio*.

d *95% Confidence Interval*.

#### Dispensing of Gastrointestinal Nutraceuticals

A number of clinical signs including diarrhoea with blood (OR 0.7, 95% CI 0.6–0.9), vomiting with (OR 0.3, 95% CI 0.2–0.5) or without blood (OR 0.6, 95% CI 0.5–0.7), body temperature between 39.5 and 39.9°C (OR 0.5, 95% CI 0.3–0.8), other clinical signs (OR 0.4, 95% CI 0.2–0.7), and moderate and severe cases (OR 0.6, 95% CI 0.5–0.8) were all less frequently associated with a gastrointestinal nutraceutical being dispensed (Table 6). However, a mixed diarrhoeic pattern was associated with increased odds (OR 1.33, 95% CI 1.04–1.71). Odds decreased with age to approximately 4 years of age and remained broadly static until 10 years of age, before decreasing further (Figure 2A). Univariable results are available in Supplementary Table 7. A cubic polynomial term was included to model age at consultation; no interaction terms significantly improved the fit of the model.

### Analysis of Longitudinal Outcomes

#### Cases Lost to Follow-Up

In total, 754 cases (23.6% of total cases) were lost to follow-up. Currently insured (OR 0.7, 95% CI 0.5–0.9), recently vaccinated (OR 0.3. 95% CI 0.3–0.4) or neutered (OR 0.4, 95% CI 0.3–0.5) dogs had lower odds of being lost to follow-up (Table 7). Increasing age was associated with increased probability of a case being lost to follow-up until approximately 4 years of age, decreasing slightly between four and twelve, before increasing once more (Figure 2B). Univariable results are available in Supplementary Table 8. A cubic polynomial term was included to model age at consultation; no interaction terms significantly improved the fit of the model.

**Table 7:** Parameter estimates from a finalised mixed effects logistic regression model, modelling on a case-level loss to follow-up and 10 day diarrhoea resolution against a range of risk factors.

**Random effect**	**Variance**	**SD^**a**^**	**Variable**	**Category**	**β**	**SE^**b**^**	**OR^**c**^ (95% CI)^**d**^**	***P***
**LOSS TO FOLLOW-UP**								
Practice	0.42	0.65		Intercept	0.29	0.16	1.34 (0.98–1.82)	
Site	0.24	0.49	Diarrhoea	Without blood	-	-	1.0	-
				With blood	0.15	0.10	1.16 (0.96–1.40)	0.13
			Insurance status	Uninsured	-	-	1.0	-
				**Insured**	**−0.36**	**0.13**	**0.70 (0.54–0.89)**	**<0.01**
			Neutered status	Unneutered	-	-	1.0	-
				**Neutered**	**−0.88**	**0.10**	**0.41 (0.34–0.51)**	**<0.01**
			Vaccination status	Unvaccinated	-	-	1.0	-
				**Vaccinated**	**−1.16**	**0.10**	**0.32 (0.26–0.39)**	**<0.01**
			Gastrointestinal agent	Not prescribed	-	-	1.0	-
				**Prescribed**	**0.32**	**0.10**	**1.38 (1.14–1.66)**	**<0.01**
			Age (years)	Age—linear	−0.09	0.09	0.92 (0.77–1.10)	0.35
				**Age—quadratic**	**−0.38**	**0.09**	**0.69 (0.58–0.82)**	**<0.01**
				**Age—cubic**	**0.20**	**0.05**	**1.22 (1.11–1.35)**	**<0.01**
**DIARRHOEA RESOLUTION**								
Practice	0.04	0.19		Intercept	3.50	0.29	33.00 (18.50–58.80)	
Site	0.07	0.26	Vomit	No vomit	-	-	1.0	-
				**Without blood**	**0.45**	**0.23**	**1.58 (1.01–2.46)**	**0.05**
				With blood	−0.87	0.47	0.42 (0.17–1.05)	0.06
			Diarrhoeic pattern	Large intestinal	-	-	1.0	-
				**Mixed**	**−0.57**	**0.28**	**0.57 (0.33–0.98)**	**0.04**
				Small intestinal	−0.25	0.24	0.78 (0.48–1.25)	0.30
				**Unknown**	**−0.78**	**0.32**	**0.46 (0.25–0.85)**	**0.01**
			Diet advice + GI nutraceutical alone	Not dispensed	-	-	1.0	-
				**Dispensed**	**1.03**	**0.40**	**2.79 (1.27–6.12)**	**0.01**
			Age (years)	Age—linear	−0.33	0.19	0.72 (0.50–1.04)	0.08
				**Age—quadratic**	**−0.44**	**0.19**	**0.65 (0.44–0.94)**	**0.02**
				Age—cubic	0.20	0.11	1.22 (0.98–1.52)	0.08

*Bold values are indicate significant findings*.

a *Standard Deviation*.

b *Standard Error*.

c *Odds Ratio*.

d *95% Confidence Interval*.

#### Diarrhoea Resolution

Cases euthanised on initial presentation (*n* = 6) and lost to follow-up (*n* = 754) were excluded, leaving 2,429 cases available for resolution analyses. By the 10^th^ day following initial presentation, 95.4% (95% CI 94.5–96.3) of cases were considered resolved; 7.6% of resolved cases were recorded as such in the clinical narrative, the remaining cases were assumed to be resolved by the absence of any further gastrointestinal-related consultations between 11 and 30 days following initial presentation.

Univariable analyses are available in Supplementary Table 9. Dogs presenting with a mixed (OR 0.57, 95% CI 0.33–0.98) or unknown (OR 0.46, 95% CI 0.25–0.85) diarrhoeic pattern were less frequently resolved (Table 7). Though no pharmaceutical agent prescribed exclusively were associated with significant variant odds of resolution, when owners were provided with dietary modification advice combined with gastrointestinal nutraceuticals but no other therapy, such cases had increased odds of resolution by 10 days post initial presentation (OR 2.8, 95% CI 1.3–6.1). This latter finding was also observed when only mild, normothermic (<39.5°C), non-haemorrhagic cases were modelled (data not presented). There was little variation in probability of resolution and age (Figure 2B). A cubic polynomial term was included to model age at consultation; no interaction terms significantly improved the fit of the model.

## Discussion

Canine acute diarrhoea is a frequent cause of presentation to primary veterinary practice (5); a range of aetiologies are associated with diarrhoea (6), a minority of which can be life threatening (9). When cases are first presented, practitioners need to make complex decisions around case management, often in the absence of any specific diagnosis (5). There is a need to understand these choices and to explore new ways of evidencing their effect, particularly in the context of systemic antimicrobial prescription. Here we used EHRs collected from a large number of veterinary practices, supplemented by structured questionnaire responses, to describe clinical signs exhibited by dogs with acute diarrhoea, characterise common management and treatment strategies, and assess the outcome of cases observed longitudinally.

This study represented the first attempt to harness overall veterinary-assessed opinion of case severity, with the vast majority being described as mild (83.6%). Only 17 cases were classed as severe, with six of these being euthanised on initial presentation. Whilst this limited our ability to describe severe disease, our findings further confirmed diarrhoea as primarily a mild condition in dogs (1). In this study, the majority of cases presented with non-vomiting, non-haemorrhagic diarrhoea, broadly consistent with previous studies (2, 5). However, diarrhoea with blood (41% of cases) and vomiting (36% of cases) was more common than previously described (25 and 18%, respectively) (5). This previous study considered all cases of diarrhoea regardless of clinical sign duration, also observing “uncomplicated diarrhea” (absence of vomiting or haemorrhagic diarrhoea) to be more common in cases of longer disease duration. This suggests that the presence of clinical signs potentially alarming to owners' e.g., haemorrhagic diarrhoea, might prompt these owners to seek veterinary attention more rapidly, potentially explaining the higher prevalence of such signs recorded here in acute cases.

Diagnostic tests were rarely used in this population (9% of all cases), and less commonly than previously reported (3, 5). This might again reflect the primary presentation nature of this study, and the generally mild nature of the reported disease. Hence, it can be reasonably assumed that most prescriptions described in this population were empirical, particularly considering that the majority were provided at initial presentation rather than over the following 10 days. Medical prescribers often perceive pressure to implement a material management plan (30) which may lead to unnecessary prescriptions, including those for antimicrobials (31); it is possible that such pressures might also influence veterinary prescription decisions (32). Diagnostic investigation should take place if an infectious aetiology is suspected (33). Unfortunately, it was not possible to determine the number of cases where the consulting veterinary professional suspected such an infectious aetiology in this study; such analyses could be of considerable future value, particularly in relation to antimicrobial stewardship.

Presence of blood in diarrhoea was significantly associated with increased odds of a systemic antimicrobial prescription being provided. This has been previously observed (5), and likely reflects a perception of increased bacteraemia risk (33). However, there is increasing evidence to suggest antimicrobial therapy is not required in such cases (9, 10, 15, 23), with a recent study finding a significant proportion of canine AHDS patients fulfilling clinical bacteraemia criteria actually tested negative on blood culture (9). Odds of a systemic antimicrobial prescription were also increased for all body temperature categories exceeding 39.0°C. Of note, body temperature was inconsistently recorded, revealing a limitation of clinical narrative analyses. Nevertheless, our findings suggest differences of opinion as to what body temperature would indicate presence or high risk of bacteraemia. Although pyrexia has been defined as body temperature in excess of 39.7°C (34), previous studies focusing on diarrhoea have variably defined pyrexia/hyperthermia between 38.8 and 39.5°C (2, 22), even altering definition by dog size (9). This study identified that 35.7% of normothermic (under 39.5°C), mild, non-haemorrhagic cases (*n* = 1,050) still prescribed systemic antimicrobials at initial presentation. On this evidence, it would thus seem that our study has identified a reasonable proportion of cases not at clear risk of sepsis treated with systemic antimicrobials regardless, in contravention to current prescribing guidance^5^. Hence, establishing a consistent definition of sepsis risk may be of some importance for effective antimicrobial stewardship. Assisting practitioner identification of patients at risk of sepsis remains a challenge across veterinary and medical care (35). In the absence of a specific diagnosis, clinical scoring has previously been successfully utilised to uniformly measure clinical severity and response to therapy (9, 10, 22). It could be of value to define more universal indicators of sepsis, and to investigate the potential benefit which could be gained, both epidemiologically and practically, from routinely applying such methods in first opinion practice.

The most frequently prescribed systemic antimicrobial in this study was metronidazole, consistent with previous studies (5, 21). This finding also suggests that the predominant concern of the prescribing veterinary surgeon is treatment of anaerobic bacterial species e.g., *Clostridium perfringens*, though the causative role of such bacteria in gastrointestinal disease has recently been brought into question (16). Further, current prescribing guidance recommends metronidazole use for chronic diarrhoea/chronic enteropathy treatment trials alone once all other diagnostic test and empirical treatment options have been exhausted (36), again suggesting limited compliance with existing guidance. In total, systemic antimicrobials were prescribed to 50% of cases, comparable or lower than previously described (46.5, 63, and 71%) (2, 5, 7). We have recently identified an approximately 30% reduction in systemic antimicrobial prescription in consultations for gastrointestinal disease between 2014 and 2018 (and a simultaneous approximately 25% increase in gastrointestinal nutraceutical prescription frequency) (37). Though it was not possible to observe a direct change in management approach by individual veterinary surgeons as repeated measures per surgeon were not recorded, our findings here could suggest that the manner with which veterinary surgeons manage gastroenteric disease and acute canine diarrhoea is changing. However, a prospective cohort study might be better placed to demonstrate this more definitively. If present, this finding might reflect increased awareness of voluntary prescribing guidance recommending antimicrobial therapy to be reserved only for acute diarrhoea cases exhibiting, or at risk, of bacteraemia or sepsis. We further recognise the opportunities afforded by providing prescription benchmarking statistics to practitioners, enabling them to effectively reflect on their own decision-making and consider changing as a result. Indeed this is an area of active development for us currently through projects such as “mySavsnetAMR;” all practices participating in SAVSNet also enjoy free access to a secure, anonymised benchmarking website for this purpose (38).

In contrast to systemic antimicrobial prescription, gastrointestinal nutraceutical dispensing frequency was considerably greater than in a previous study (61% of cases compared to 26%) (2). Study methodological differences accepted, it has been suggested that gastroenteric nutraceuticals may form a “no harm” alternative to antibiosis in order to effectively manage owner treatment plan expectations (32). Of further interest, our findings suggest that a combination of dietary modification and gastrointestinal nutraceuticals without prescription of any studied pharmaceutical agent could aid resolution of diarrhoeic clinical signs. Though evidence remains scarce, previous studies have suggested that probiotics might be efficacious in ameliorating infectious, non-infectious or idiopathic diarrhoea in dogs (25); it is possible that we might be observing such an effect here. However, since we have not randomised cases into treatment groups, there remains a possibility of bias according to over-simplification of clinical severity scoring as used here (39). Therefore, whilst evidence of *in vivo* efficacy of gastrointestinal nutraceuticals remains limited (25) we advocate some continued caution over wholeheartedly embracing nutraceutical use. As with all other areas of veterinary practice, clear clinical evidence when available should drive decision making, and when unavailable efforts should be made to fill such gaps in knowledge. As such, we believe the field is now ready for a fully randomised pragmatic trial to provide more definitive evidence surrounding the clinical benefit (or absence thereof) of prescribing antimicrobials and other agents to manage acute canine diarrhoea.

Regarding endoparasiticides/endectocides, weight loss was significantly associated with increased odds of prescription, possibly reflecting the view that weight loss is often associated with parasitic infection (33). It should be remembered that some endoparasiticides/endectocides^6^ (as well as gastrointestinal nutraceuticals) are available without the need of a prescription such that it is likely we have under-estimated the actual use of these agents in this study. Similarly, this study focused on in-consultation prescription decisions; expanding its scope to include EHRs for referred animals and in-patient (hospitalised) records would more completely represent all aspects of companion animal practice.

The effect of the animal's age on odds of pharmaceutical prescription were complex and could be separated into two groups: systemic antimicrobials or gastrointestinal agents were prescribed more commonly to older animals, whereas endoparasiticides/endectocides or gastrointestinal nutraceuticals were prescribed more commonly to younger animals, possibly reflecting increased parasitic or viral infection in puppies (33, 40). On univariable analyses, odds of a case being considered moderate or severe did increase with age (data not presented); however, including severity as an interaction with age did not improve the fit of the model.

Classical approaches to defining the benefit of particular treatments is to use randomised control trials, systematic reviews or meta-analyses. For canine gastroenteritis treated in primary care, trials of any form whether randomised or not are limited in number, and generally small in size (9, 10, 22), such that there is a dearth of evidence with which practitioners can base their treatment choices. One route to increasing evidence and complementing the highest level data from trials are pragmatic and observational studies using EHRs collected at scale (41). Here, our observational approach suggests no clear link between any therapy choice and outcome; a finding corroborated for antimicrobial therapy by earlier smaller studies (9, 10, 22). There is an increasing pressure on both medical and veterinary prescribers to make responsible therapeutic decisions, reflecting best available clinical evidence (19), and our findings would appear to broadly support the view that using antimicrobials for management of acute diarrhoea is largely unnecessary (10).

Whilst the purely observational approach used here was useful, this study was limited by nearly a quarter of cases being lost to follow-up. Without specific intervention, it is impossible to determine whether these cases simply recovered, moved to another veterinary practice, opted out of further SAVSNET participation, or died. However, we did show insured, neutered, or vaccinated dogs to be associated with significantly decreased odds of being lost to follow-up, suggesting either owners of such dogs are more likely to engage with regular veterinary care, or their vets are more likely to request follow up consultations. Similarly, the odds of being lost to follow-up also broadly increased as an animal's age increased. Whilst this might represent increased odds of death (42), it might also suggest that as owners become more experienced with their pet, they are less likely to re-present with their dog when investigating/treating disease.

Defining outcome in an observational study of this type presents certain challenges. When reviewing cases that re-presented within 10 days of initial presentation, dogs often re-presented at the request of the veterinary surgeon, or re-presented for an unrelated complaint (data not presented). We therefore concluded that time between initial and subsequent presentations alone to be an unreliable measure of clinical resolution and response to treatment. Thus, we used a 10 day period as a broad representation of the acute diarrhoea therapy period (9, 10, 22, 33), subsequently using MPC between 11 and 30 days as an indicator of gastroenteric clinical sign persistence or re-emergence. Though seemingly appropriate, loss to follow-up limited our ability to fully characterise clinical resolution. In addition, considerable therapeutic management diversity was seen; this represents a significant challenge when seeking to define the effect of each pharmaceutical intervention which would only be compounded if we had also considered additional factors such as dosage or course length. Here we focused on five pharmaceutical classes commonly prescribed to diarrhoea cases (2); other pharmaceutical classes were prescribed which might also have had an impact on clinical resolution. Considering these limiting factors, a more structured approach, including contacting owners after initial presentation, could complement the more observational approach taken here.

The issues posed by veterinary surgeons failing to record, or variably recording information within the clinical narrative has been previously noted (7). We also observed such difficulties (e.g., body temperature recording), though we found combining compulsory randomised questionnaire data with the EHR to at least partially mitigate this issue (e.g., case severity). To encourage engagement, the questionnaire was only deployed in a small proportion of randomly selected relevant consultations; the cases studied here therefore only represent a small percentage of cases available within the SAVSNET database. It should also be remembered that questionnaire responses were self-defined; individual variation in case definition is therefore possible. As text mining capabilities advance (7), the confidence with which we could identify and follow cases using such approaches is likely to increase, potentially unlocking a considerably greater number of cases for analyses. However, in the mean time we would advocate the use of a combined textual and questionnaire response analysis as demonstrated here.

## Conclusions

This study successfully demonstrated the ability of combined structured, semi-structured and unstructured data to characterise factors associated with pharmaceutical prescription in acute canine diarrhoea cases. Not surprisingly, we saw considerable therapeutic diversity between cases, a number of which contradicted current prescribing guidance. Considering the threat posed by antimicrobial resistance especially, this suggests that latest clinical evidence is not effectively being disseminated throughout the profession. The findings presented here complement other studies, and suggests that efforts should be re-doubled to effectively disseminate latest clinical evidence to the wider, and particularly first opinion, veterinary profession. Though future methodological improvements are recommended, this study broadly supports the view that systemic antimicrobials are largely unnecessary in acute diarrhoea cases. The only intervention positively associated with resolution odds was provision of dietary modification advice and gastrointestinal nutraceuticals; hence we would urgently recommend further work exploring the precise impact of prebiotics, probiotics etc. on gastrointestinal health in our canine population.

## Data Availability

The raw data supporting the conclusions of this manuscript will be made available by the authors, without undue reservation, to any qualified researcher.

## Author Contributions

DS devised study design, analysed data, and drafted the manuscript, supervised by PJ, PN, GP, and AR. NW is a co-investigator on the grant funding this work, and assisted with revising the manuscript. FS-V and SD are co-investigators of the wider SAVSNET project, assisting with revising the manuscript.

### Conflict of Interest Statement

The authors declare that the research was conducted in the absence of any commercial or financial relationships that could be construed as a potential conflict of interest.

## Footnotes

^1^AOD Package. Available from: https://cran.r-project.org/package=aod

^2^LME4 Package. Available from: https://cran.r-project.org/package=lme4

^3^CAR Package. Available from: https://cran.r-project.org/package=car.

^4^sjPlot Package. Available from: https://cran.r-project.org/package=sjPlot

^5^PROTECT ME. Available from: https://www.bsava.com/Resources/Veterinary-resources/PROTECT-ME

^6^Veterinary Medicines Directorate product information database. Available from: www.vmd.defra.gov.uk/ProductInformationDatabase/Default.aspx
