# Supplementary material for: Pharmaceutical Prescription in Canine Acute Diarrhoea: A Longitudinal Electronic Health Record Analysis of First Opinion Veterinary Practices
Source: Front Vet Sci. 2019 Jul 2;6:218. doi: 10.3389/fvets.2019.00218 (PMC6615257; doi:10.3389/fvets.2019.00218)
Supplement: Supplementary file 1 [file Data_Sheet_1.docx]

**Supplementary material**

**Table 1**

Number and relative percentage of pharmaceutical classes of systemic antimicrobials; systemic anti-inflammatories; gastrointestinal agents, and endoparasiticides / endectocides most frequently prescribed at initial diarrhoea presentation and over the subsequent 10 days.

| **Pharmaceutical class(s)** | ***n* prescribing cases** | **% prescription (CI) ^a^** |
| --- | --- | --- |
| **Systemic antimicrobial (n cases = 1642)** | | |
| Metronidazole | 788 | 47.0 (41.0-53.1) |
| Clavulanic acid potentiated amoxicillin | 380 | 22.7 (19.3-26.1) |
| Amoxicillin | 203 | 12.1 (8.7-15.6) |
| Metronidazole and spiramycin | 108 | 6.5 (3.9-9.1) |
| Amoxicillin and metronidazole | 64 | 3.8 (0.3-7.3) |
| Clavulanic acid potentiated amoxicillin and metronidazole | 39 | 2.3 (1.5-3.2) |
| Clavulanic acid potentiated amoxicillin and amoxicillin | 29 | 1.7 (0.9-2.5) |
| Fluoroquinolone | 12 | 0.7 (0.1-1.3) |
| Macrolide | 10 | 0.6 (0.0-1.8) |
| Potentiated sulphonamide | 9 | 0.5 (0.2-0.9) |
| **Systemic anti-inflammatory (n cases = 489)** | | |
| Glucocorticoid | 399 | 81.3 (73.6-89.1) |
| Oxicam | 69 | 14.1 (6.9-21.3) |
| Coxib | 7 | 1.5 (0.3-2.6) |
| Profen | 5 | 1.0 (0.1-2.0) |
| Oclacitinib | 4 | 0.8 (0.0-1.7) |
| Glucocorticoid and oxicam | 2 | 0.4 (0.0-1.0) |
| Disease modifying osteoarthritis drug | 1 | 0.2 (0.0-0.6) |
| Disease modifying osteoarthritis drug and glucorticoid | 1 | 0.2 (0.0-0.6) |
| Glucocorticoid and profen | 1 | 0.2 (0.0-0.6) |
| **Gastrointestinal agent (n cases = 1187)** | | |
| Maropitant | 554 | 44.6 (39.9-49.3) |
| H_2_ histamine receptor antagonist | 259 | 20.8 (16.8-24.8) |
| Maropitant and H_2_ histamine receptor antagonist | 199 | 16.0 (13.3-18.7) |
| Metoclopramide | 43 | 3.5 (2.0-4.9) |
| Proton pump inhibitor | 39 | 3.1 (1.9-4.3) |
| Sulfasalazine | 39 | 3.1 (1.4-4.8) |
| Maropitant and proton pump inhibitor | 34 | 2.7 (1.6-3.9) |
| Clay | 12 | 1.0 (0.4-1.6) |
| Maropitant and sulfasalazine | 8 | 0.6 (0.1-1.2) |
| H_2_ histamine receptor antagonist and proton pump inhibitor | 6 | 0.5 (0.1-0.8) |
| **Endoparasiticide / endectocide (n cases = 304)** | | |
| Milbemycin and quinoline | 146 | 48.0 (41.5-54.5) |
| Benzimidazole | 72 | 23.6 (17.8-29.5) |
| Milbemycin | 39 | 12.8 (8.8-16.8) |
| Benzimidazole, quinoline and tetrahydropyrmidine | 21 | 6.9 (3.5-10.3) |
| Quinoline | 15 | 4.9 (2.4-7.5) |
| Benzimidazole and milbemycin | 8 | 2.6 (0.6-4.4) |
| Avermectin | 1 | 0.3 (0.0-1.0) |
| Benzimidazole, milbemycin and quinoline | 1 | 0.3 (0.0-1.0) |
| Benzimidazole, quinoline, milbemycin and tetrahydropyrmidine | 1 | 0.3 (0.0-1.0) |

^a^ Percentage of total prescribing cases within pharmaceutical family, 95% confidence interval

**Table 2:** Descriptive summary of pharmaceutical prescriptions and dispensing of nutraceutical products both at initial presentation and when the subsequent nine days (inclusive) post-presentation were considered. Longitudinal outcome is also displayed, with all comparisons shown when considered by presence of clinical signs at initial presentation. D+H-V- = presence of diarrhoea without blood and non-vomiting; D+H+V- = presence of diarrhoea with blood and non-vomiting; D+H-V+ = presence of diarrhoea without blood and vomiting; D+H+V+ = presence of diarrhoea with blood and vomiting. Vomiting is inclusive of presence or absence of blood in vomit.

| **Category** | **D+H-V-**  **(*n* = 1150 cases)** | **D+H+V-**  **(*n* = 891 cases)** | **D+H-V+**  **(*n* = 743 cases)** | **D+H+V+**  **(*n* = 405 cases)** |
| --- | --- | --- | --- | --- |
|  | **% (95% CI) ^a^** | **% (95% CI)** | **% (95% CI)** | **% (95% CI)** |
| **Therapy – initial presentation** | | | | |
| Pharmaceutical agent | 64.1 (60.5-67.8) | 82.2 (79.3-85.0) | 89.5 (87.0-91.9) | 90.1 (87.1-93.2) |
| Systemic antimicrobial | 40.1 (35.4-44.7) | 65.4 (61.5-69.2) | 37.5 (32.8-42.3) | 64.6 (58.9-70.4) |
| Systemic anti-inflammatory | 13.1 (9.6-16.6) | 17.0 (12.6-21.5) | 12.3 (7.3-17.3) | 14.5 (9.2-19.8) |
| Gastrointestinal agent | 15.3 (12.7-17.9) | 19.4 (16.4-22.5) | 76.0 (72.3-79.7) | 70.6 (65.9-75.4) |
| Endoparasiticide and / or endectocide | 10.1 (8.1-12.1) | 9.4 (7.4-11.5) | 3.9 (2.5-5.3) | 5.2 (2.8-7.6) |
| Gastrointestinal nutraceutical | 69.0 (64.4-73.7) | 59.8 (55.0-64.5) | 55.4 (50.4-60.4) | 50.1 (44.7-55.5) |
| Euthanasia / death | 0.2 (0.0-0.4) | 0.1 (0.0-0.3) | 0.3 (0.0-0.6) | 0.2 (0.0-0.7) |
| **Therapy – initial presentation and / or within 10 days of initial presentation** | | | | |
| Pharmaceutical agent | 68.9 (65.5-72.3) | 83.8 (81.2-86.5) | 91.6 (89.5-93.8) | 92.1 (89.4-94.8) |
| Systemic antimicrobial | 42.9 (38.4-47.5) | 67.4 (63.6-71.2) | 41.1 (36.7-46.6) | 67.7 (62.1-73.2) |
| Systemic anti-inflammatory | 14.2 (10.7-17.7) | 17.7 (13.3-22.1) | 13.1 (8.3-18.0) | 17.0 (11.7-22.3) |
| Gastrointestinal agent | 17.4 (14.6-20.2) | 20.5 (17.3-23.7) | 76.6 (73.0-80.3) | 71.6 (66.9-76.3) |
| Endoparasiticide and / or endectocide | 12.0 (9.9-14.1) | 11.6 (9.2-13.9) | 5.4 (3.7-7.0) | 5.6 (3.1-8.2) |
| Gastrointestinal nutraceutical | 69.6 (65.0-74.1) | 60.4 (55.6-65.3) | 56.7 (51.8-61.6) | 52.1 (46.6-57.7) |
| Euthanasia / death | 0.3 (0.0-0.6) | 0.3 (0.0-0.7) | 0.7 (0.1-1.2) | 0.2 (0.0-0.7) |
| **Outcome** | | | | |
| Resolution (10 day) | 75.1 (72.1-78.2) | 70.9 (67.3-74.5) | 71.7 (67.9-75.6) | 71.1 (66.8-75.4) |
| Lost to follow-up | 21.1 (18.0-24.2) | 25.0 (21.4-28.5) | 25.2 (21.4-28.9) | 24.9 (20.7-29.2) |

^a^ Percentage of cases (95% confidence interval)

**Table 3**

Systemic anti-inflammatories: Parameter estimates from a series of univariable mixed effects logistic regression models, modelling on a case-level the outcome variable ‘presence of systemic anti-inflammatory prescription’ against a number of categorical and continuous risk factors.

| **Variable** | **Category** | **β** | **SE ^a^** | **OR ^b^** | **Lower CI ^c^** | **Upper CI** | ***P*** |
| --- | --- | --- | --- | --- | --- | --- | --- |
| Insurance status | Uninsured (Intercept) | -11.96 | 0.43 | 0.00 | 0.00 | 0.00 |  |
|  | Insured | -0.27 | 0.65 | 0.76 | 0.21 | 2.72 | 0.68 |
| Sex | Female (Intercept) | -11.96 | 0.48 | 0.00 | 0.00 | 0.00 |  |
|  | Male | -0.15 | 0.52 | 0.86 | 0.31 | 2.41 | 0.78 |
| Neutered status | Un-neutered (Intercept) | -11.89 | 0.51 | 0.00 | 0.00 | 0.00 |  |
|  | Neutered | -0.20 | 0.54 | 0.82 | 0.28 | 2.37 | 0.71 |
| Vaccination status | Unvaccinated (Intercept) | -12.15 | 0.60 | 0.00 | 0.00 | 0.00 |  |
|  | Vaccinated | 0.16 | 0.61 | 1.17 | 0.36 | 3.87 | 0.79 |
| Microchip status | Un-microchipped (Intercept) | -11.99 | 0.51 | 0.00 | 0.00 | 0.00 |  |
|  | Microchipped | -0.07 | 0.53 | 0.94 | 0.34 | 2.62 | 0.90 |
| Diarrhoea | Without blood (Intercept) | -12.28 | 0.49 | 0.00 | 0.00 | 0.00 |  |
|  | With blood | 0.54 | 0.52 | 1.72 | 0.63 | 4.72 | 0.29 |
| Vomit | Absent (Intercept) | -11.86 | 0.00 | 0.00 | 0.00 | 0.00 |  |
|  | Without blood | 0.09 | 0.42 | 1.09 | 0.48 | 2.48 | 0.84 |
|  | With blood | -3.86 | 0.00 | 0.02 | 0.02 | 0.02 | <0.01 |
| Other clinical signs | Absent (Intercept) | -12.04 | 0.38 | 0.00 | 0.00 | 0.00 |  |
|  | Present | 0.55 | 1.70 | 1.73 | 0.06 | 48.00 | 0.75 |
| Poor appetite | Absent (Intercept) | -11.96 | 0.41 | 0.00 | 0.00 | 0.00 |  |
|  | Present | -0.55 | 0.88 | 0.58 | 0.10 | 3.24 | 0.53 |
| Weight loss | Absent (Intercept) | -12.02 | 0.39 | 0.00 | 0.00 | 0.00 |  |
|  | Present | -0.35 | 2.77 | 0.71 | 0.00 | 160.45 | 0.90 |
| Melaena | Absent (Intercept) | -12.03 | 0.39 | 0.00 | 0.00 | 0.00 |  |
|  | Present | 0.12 | 3.92 | 1.12 | 0.00 | 2420.70 | 0.98 |
| Severity | Mild (Intercept) | -12.06 | 0.42 | 0.00 | 0.00 | 0.00 |  |
|  | Moderate | 0.18 | 0.65 | 1.20 | 0.33 | 4.33 | 0.78 |
| Diarrhoea pattern | Large intestinal (Intercept) | -11.18 | 1.90 | 0.00 | 0.00 | 0.00 |  |
|  | Mixed | -0.35 | 1.95 | 0.70 | 0.02 | 32.40 | 0.86 |
|  | Small intestinal | -0.68 | 1.98 | 0.51 | 0.01 | 24.57 | 0.73 |
|  | Unknown | -1.26 | 6.89 | 0.28 | 0.00 | 209419.62 | 0.86 |
| Body temperature | Normal / <39 °C (Intercept) | -12.42 | 0.50 | 0.00 | 0.00 | 0.00 |  |
|  | Not recorded | 0.94 | 0.57 | 2.56 | 0.85 | 7.77 | 0.10 |
|  | 39.0 °C ≤ 39.4 °C | 0.40 | 0.82 | 1.49 | 0.30 | 7.48 | 0.63 |
|  | 39.5 °C ≤ 39.9 °C | 0.85 | 1.25 | 2.34 | 0.20 | 27.08 | 0.50 |
|  | 40.0 °C ≤ | 1.26 | 2.02 | 3.53 | 0.07 | 185.33 | 0.53 |
| **Continuous risk factor** | | | | | | | |
| Age (years) | Intercept | -12.03 | 0.41 | 0.00 | 0.00 | 0.00 |  |
|  | Age - linear | 0.12 | 0.25 | 1.13 | 0.69 | 1.85 | 0.63 |

^a^ Standard error

^b^ Odds ratio

^c^ 95% Confidence interval

**Table 4**

Systemic antimicrobials: Parameter estimates from a series of univariable mixed effects logistic regression models, modelling on a case-level the outcome variable ‘presence of systemic antimicrobial prescription’ against a number of categorical and continuous risk factors.

| **Variable** | **Category** | **β** | **SE ^a^** | **OR ^b^** | **Lower CI ^c^** | **Upper CI** | ***P*** |
| --- | --- | --- | --- | --- | --- | --- | --- |
| Insurance status | Uninsured (Intercept) | -0.14 | 0.08 | 0.87 | 0.74 | 1.02 |  |
|  | Insured | 0.19 | 0.09 | 1.20 | 1.00 | 1.45 | 0.05 |
| Sex | Female (Intercept) | -0.12 | 0.09 | 0.89 | 0.75 | 1.05 |  |
|  | Male | 0.06 | 0.08 | 1.06 | 0.91 | 1.24 | 0.45 |
| Neutered status | Un-neutered (Intercept) | -0.14 | 0.10 | 0.87 | 0.72 | 1.05 |  |
|  | Neutered | 0.08 | 0.09 | 1.08 | 0.91 | 1.27 | 0.38 |
| Vaccination status | Unvaccinated (Intercept) | -0.10 | 0.10 | 0.90 | 0.74 | 1.10 |  |
|  | Vaccinated | 0.02 | 0.09 | 1.02 | 0.85 | 1.22 | 0.86 |
| Microchip status | Un-microchipped (Intercept) | -0.11 | 0.09 | 0.90 | 0.75 | 1.07 |  |
|  | Microchipped | 0.03 | 0.08 | 1.03 | 0.88 | 1.20 | 0.73 |
| Diarrhoea | Without blood (Intercept) | -0.63 | 0.09 | 0.53 | 0.45 | 0.64 |  |
|  | With blood | 1.32 | 0.09 | 3.76 | 3.17 | 4.45 | <0.01 |
| Vomit | Absent (Intercept) | -0.06 | 0.08 | 0.94 | 0.80 | 1.10 |  |
|  | Without blood | -0.11 | 0.08 | 0.90 | 0.76 | 1.06 | 0.21 |
|  | With blood | 0.21 | 0.27 | 1.24 | 0.74 | 2.08 | 0.42 |
| Other clinical signs | Absent (Intercept) | -0.10 | 0.08 | 0.91 | 0.78 | 1.06 |  |
|  | Present | 0.31 | 0.32 | 1.36 | 0.72 | 2.57 | 0.34 |
| Poor appetite | Absent (Intercept) | -0.11 | 0.08 | 0.90 | 0.77 | 1.05 |  |
|  | Present | 0.10 | 0.11 | 1.11 | 0.89 | 1.39 | 0.36 |
| Weight loss | Absent (Intercept) | -0.10 | 0.08 | 0.91 | 0.78 | 1.05 |  |
|  | Present | 0.53 | 0.37 | 1.69 | 0.82 | 3.50 | 0.16 |
| Melaena | Absent (Intercept) | -0.09 | 0.08 | 0.91 | 0.79 | 1.06 |  |
|  | Present | -0.16 | 0.61 | 0.85 | 0.26 | 2.80 | 0.79 |
| Severity | Mild (Intercept) | -0.17 | 0.08 | 0.84 | 0.72 | 0.99 |  |
|  | Moderate | 0.47 | 0.11 | 1.60 | 1.30 | 1.98 | <0.01 |
| Diarrhoea pattern | Large intestinal (Intercept) | 0.15 | 0.09 | 1.16 | 0.97 | 1.39 |  |
|  | Mixed | -0.15 | 0.12 | 0.86 | 0.68 | 1.08 | 0.20 |
|  | Small intestinal | -0.42 | 0.09 | 0.66 | 0.55 | 0.79 | <0.01 |
|  | Unknown | -0.83 | 0.16 | 0.43 | 0.32 | 0.59 | <0.01 |
| Body temperature | Normal / <39 °C (Intercept) | -0.25 | 0.09 | 0.78 | 0.66 | 0.92 |  |
|  | Not recorded | 0.14 | 0.10 | 1.15 | 0.95 | 1.39 | 0.15 |
|  | 39.0 °C ≤ 39.4 °C | 0.49 | 0.12 | 1.63 | 1.28 | 2.07 | <0.01 |
|  | 39.5 °C ≤ 39.9 °C | 1.42 | 0.24 | 4.13 | 2.57 | 6.63 | <0.01 |
|  | 40.0 °C ≤ | 1.05 | 0.44 | 2.85 | 1.20 | 6.75 | 0.02 |
| **Continuous risk factor** | | | | | | | |
| Age (years) | Intercept | 0.16 | 0.10 | 1.17 | 0.97 | 1.41 |  |
|  | Age - linear | 0.21 | 0.08 | 1.24 | 1.07 | 1.43 | 0.01 |
|  | Age - quadratic | -0.31 | 0.08 | 0.73 | 0.63 | 0.85 | <0.01 |
|  | Age - cubic | 0.10 | 0.04 | 1.11 | 1.02 | 1.21 | 0.02 |

^a^ Standard error

^b^ Odds ratio

^c^ 95% Confidence interval

**Table 5**

Gastrointestinal agents: Parameter estimates from a series of univariable mixed effects logistic regression models, modelling on a case-level the outcome variable ‘presence of gastrointestinal agent prescription’ against a number of categorical and continuous risk factors.

| **Variable** | **Category** | **β** | **SE ^a^** | **OR ^b^** | **Lower CI ^c^** | **Upper CI** | ***P*** |
| --- | --- | --- | --- | --- | --- | --- | --- |
| Insurance status | Uninsured (Intercept) | -0.58 | 0.06 | 0.56 | 0.50 | 0.63 |  |
|  | Insured | 0.19 | 0.09 | 1.20 | 1.01 | 1.43 | 0.04 |
| Sex | Female (Intercept) | -0.46 | 0.07 | 0.63 | 0.56 | 0.72 |  |
|  | Male | -0.15 | 0.08 | 0.86 | 0.74 | 1.00 | 0.05 |
| Neutered status | Un-neutered (Intercept) | -0.65 | 0.08 | 0.52 | 0.45 | 0.61 |  |
|  | Neutered | 0.17 | 0.08 | 1.19 | 1.01 | 1.40 | 0.03 |
| Vaccination status | Unvaccinated (Intercept) | -0.50 | 0.08 | 0.61 | 0.51 | 0.71 |  |
|  | Vaccinated | -0.03 | 0.09 | 0.97 | 0.82 | 1.15 | 0.70 |
| Microchip status | Un-microchipped (Intercept) | -0.59 | 0.07 | 0.55 | 0.48 | 0.63 |  |
|  | Microchipped | 0.12 | 0.08 | 1.12 | 0.97 | 1.31 | 0.13 |
| Diarrhoea | Without blood (Intercept) | -0.46 | 0.06 | 0.63 | 0.56 | 0.71 |  |
|  | With blood | -0.17 | 0.08 | 0.84 | 0.72 | 0.98 | 0.03 |
| Vomit | Absent (Intercept) | -1.74 | 0.09 | 0.18 | 0.15 | 0.21 |  |
|  | Without blood | 2.89 | 0.11 | 18.00 | 14.53 | 22.28 | <0.01 |
|  | With blood | 3.59 | 0.35 | 36.33 | 18.45 | 71.54 | <0.01 |
| Other clinical signs | Absent (Intercept) | -0.53 | 0.05 | 0.59 | 0.53 | 0.65 |  |
|  | Present | 0.26 | 0.29 | 1.30 | 0.73 | 2.31 | 0.38 |
| Poor appetite | Absent (Intercept) | -0.75 | 0.06 | 0.47 | 0.42 | 0.53 |  |
|  | Present | 1.53 | 0.12 | 4.61 | 3.67 | 5.78 | <0.01 |
| Weight loss | Absent (Intercept) | -0.54 | 0.05 | 0.59 | 0.53 | 0.65 |  |
|  | Present | 0.60 | 0.34 | 1.82 | 0.93 | 3.55 | 0.08 |
| Melaena | Absent (Intercept) | -0.53 | 0.05 | 0.59 | 0.53 | 0.65 |  |
|  | Present | 0.64 | 0.58 | 1.90 | 0.61 | 5.93 | 0.27 |
| Severity | Mild (Intercept) | -0.73 | 0.06 | 0.48 | 0.43 | 0.54 |  |
|  | Moderate | 1.17 | 0.10 | 3.21 | 2.62 | 3.94 | <0.01 |
| Diarrhoea pattern | Large intestinal (Intercept) | -0.88 | 0.07 | 0.42 | 0.36 | 0.48 |  |
|  | Mixed | 0.76 | 0.11 | 2.14 | 1.72 | 2.67 | <0.01 |
|  | Small intestinal | 0.51 | 0.09 | 1.67 | 1.40 | 1.99 | <0.01 |
|  | Unknown | 0.47 | 0.14 | 1.61 | 1.21 | 2.13 | <0.01 |
| Body temperature | Normal / <39 °C (Intercept) | -0.40 | 0.06 | 0.67 | 0.59 | 0.76 |  |
|  | Not recorded | -0.58 | 0.07 | 0.56 | 0.47 | 0.68 | <0.01 |
|  | 39.0 °C ≤ 39.4 °C | 0.02 | 0.12 | 1.02 | 0.82 | 1.28 | 0.83 |
|  | 39.5 °C ≤ 39.9 °C | 0.05 | 0.21 | 1.05 | 0.70 | 1.58 | 0.81 |
|  | 40.0 °C ≤ | -0.32 | 0.40 | 0.73 | 0.33 | 1.59 | 0.42 |
| **Continuous risk factor** | | | | | | | |
| Age (years) | Intercept | -0.25 | 0.08 | 0.78 | 0.67 | 0.90 |  |
|  | Age - linear | 0.04 | 0.07 | 1.04 | 0.91 | 1.20 | 0.58 |
|  | Age - quadratic | -0.36 | 0.07 | 0.70 | 0.60 | 0.80 | <0.01 |
|  | Age - cubic | 0.14 | 0.04 | 1.15 | 1.06 | 1.24 | <0.01 |

^a^ Standard error

^b^ Odds ratio

^c^ 95% Confidence interval

**Table 6**

Endoparasiticides / endectocides: Parameter estimates from a series of univariable mixed effects logistic regression models, modelling on a case-level the outcome variable ‘presence of endoparasiticide / endectocide prescription’ against a number of categorical and continuous risk factors.

| **Variable** | **Category** | **β** | **SE ^a^** | **OR ^b^** | **Lower CI ^c^** | **Upper CI** | ***P*** |
| --- | --- | --- | --- | --- | --- | --- | --- |
| Insurance status | Uninsured (Intercept) | -2.54 | 0.11 | 0.08 | 0.06 | 0.10 |  |
|  | Insured | -0.34 | 0.17 | 0.71 | 0.51 | 0.99 | 0.05 |
| Sex | Female (Intercept) | -2.67 | 0.12 | 0.07 | 0.06 | 0.09 |  |
|  | Male | 0.11 | 0.14 | 1.11 | 0.85 | 1.45 | 0.43 |
| Neutered status | Un-neutered (Intercept) | -2.34 | 0.13 | 0.10 | 0.08 | 0.13 |  |
|  | Neutered | -0.43 | 0.14 | 0.65 | 0.50 | 0.86 | <0.01 |
| Vaccination status | Unvaccinated (Intercept) (Intercept) | -2.17 | 0.14 | 0.11 | 0.09 | 0.15 |  |
|  | Vaccinated | -0.64 | 0.14 | 0.53 | 0.40 | 0.70 | <0.01 |
| Microchip status | Un-microchipped (Intercept) | -2.40 | 0.12 | 0.09 | 0.07 | 0.12 |  |
|  | Microchipped | -0.43 | 0.14 | 0.65 | 0.50 | 0.86 | <0.01 |
| Diarrhoea | Without blood (Intercept) | -2.63 | 0.12 | 0.07 | 0.06 | 0.09 |  |
|  | With blood | 0.05 | 0.14 | 1.05 | 0.80 | 1.37 | 0.74 |
| Vomit | Absent (Intercept) | -2.36 | 0.11 | 0.10 | 0.08 | 0.12 |  |
|  | Without blood | -0.87 | 0.17 | 0.42 | 0.30 | 0.59 | <0.01 |
|  | With blood | -1.37 | 0.73 | 0.26 | 0.06 | 1.07 | 0.06 |
| Other clinical signs | Absent (Intercept) | -2.60 | 0.10 | 0.07 | 0.06 | 0.09 |  |
|  | Present | -0.80 | 0.74 | 0.45 | 0.11 | 1.91 | 0.28 |
| Poor appetite | Absent (Intercept) | -2.60 | 0.11 | 0.08 | 0.06 | 0.09 |  |
|  | Present | -0.14 | 0.21 | 0.87 | 0.58 | 1.30 | 0.50 |
| Weight loss | Absent (Intercept) | -2.63 | 0.10 | 0.07 | 0.06 | 0.09 |  |
|  | Present | 0.80 | 0.48 | 2.22 | 0.87 | 5.64 | 0.09 |
| Melaena | Absent (Intercept) | -2.61 | 0.10 | 0.07 | 0.06 | 0.09 |  |
|  | Present | -14.91 | 60.34 | 0.00 | 0.00 | 1000< | 0.81 |
| Severity | Mild (Intercept) | -2.50 | 0.10 | 0.08 | 0.07 | 0.10 |  |
|  | Moderate | -1.05 | 0.26 | 0.35 | 0.21 | 0.58 | <0.01 |
| Diarrhoea pattern | Large intestinal (Intercept) | -2.45 | 0.13 | 0.09 | 0.07 | 0.11 |  |
|  | Mixed | -0.32 | 0.21 | 0.72 | 0.48 | 1.09 | 0.12 |
|  | Small intestinal | -0.37 | 0.16 | 0.69 | 0.50 | 0.94 | 0.02 |
|  | Unknown | -0.00 | 0.24 | 1.00 | 0.63 | 1.59 | 0.99 |
| Body temperature | Normal / <39 °C (Intercept) | -2.64 | 0.12 | 0.07 | 0.06 | 0.09 |  |
|  | Not recorded | 0.16 | 0.16 | 1.18 | 0.86 | 1.61 | 0.31 |
|  | 39.0 °C ≤ 39.4 °C | -0.05 | 0.21 | 0.95 | 0.63 | 1.44 | 0.81 |
|  | 39.5 °C ≤ 39.9 °C | -0.23 | 0.41 | 0.79 | 0.36 | 1.77 | 0.57 |
|  | 40.0 °C ≤ | -0.90 | 1.03 | 0.41 | 0.05 | 3.10 | 0.39 |
| **Continuous risk factor** | | | | | | | |
| Age (years) | Intercept | -3.17 | 0.16 | 0.04 | 0.03 | 0.06 |  |
|  | Age - linear | 0.12 | 0.18 | 1.13 | 0.80 | 1.58 | 0.50 |
|  | Age - quadratic | 0.68 | 0.14 | 1.98 | 1.52 | 2.58 | <0.01 |
|  | Age - cubic | -0.42 | 0.11 | 0.66 | 0.53 | 0.82 | <0.01 |

^a^ Standard error

^b^ Odds ratio

^c^ 95% Confidence interval

**Table 7**

Gastrointestinal nutraceuticals: Parameter estimates from a series of univariable mixed effects logistic regression models, modelling on a case-level the outcome variable ‘dispensed a gastrointestinal nutraceutical’ against a number of categorical and continuous risk factors.

| **Variable** | **Category** | **β** | **SE ^a^** | **OR ^b^** | **Lower CI ^c^** | **Upper CI** | ***P*** |
| --- | --- | --- | --- | --- | --- | --- | --- |
| Insurance status | Uninsured (Intercept) | 0.67 | 0.08 | 1.95 | 1.66 | 2.30 |  |
|  | Insured | -0.06 | 0.10 | 0.94 | 0.78 | 1.13 | 0.52 |
| Sex | Female (Intercept) | 0.61 | 0.09 | 1.85 | 1.55 | 2.20 |  |
|  | Male | 0.08 | 0.08 | 1.09 | 0.93 | 1.27 | 0.30 |
| Neutered status | Un-neutered (Intercept) | 0.69 | 0.10 | 1.98 | 1.63 | 2.41 |  |
|  | Neutered | -0.05 | 0.09 | 0.96 | 0.81 | 1.13 | 0.59 |
| Vaccination status | Unvaccinated (Intercept) | 0.56 | 0.10 | 1.75 | 1.43 | 2.15 |  |
|  | Vaccinated | 0.13 | 0.09 | 1.13 | 0.95 | 1.36 | 0.17 |
| Microchip status | Un-microchipped (Intercept) | 0.66 | 0.09 | 1.93 | 1.61 | 2.31 |  |
|  | Microchipped | -0.01 | 0.08 | 0.99 | 0.85 | 1.17 | 0.94 |
| Diarrhoea | Without blood (Intercept) | 0.78 | 0.09 | 2.19 | 1.85 | 2.59 |  |
|  | With blood | -0.32 | 0.08 | 0.73 | 0.62 | 0.85 | <0.01 |
| Vomit | Absent (Intercept) | 0.89 | 0.09 | 2.44 | 2.05 | 2.91 |  |
|  | Without blood | -0.57 | 0.09 | 0.57 | 0.48 | 0.67 | <0.01 |
|  | With blood | -1.42 | 0.28 | 0.24 | 0.14 | 0.42 | <0.01 |
| Other clinical signs | Absent (Intercept) | 0.67 | 0.08 | 1.96 | 1.68 | 2.29 |  |
|  | Present | -1.09 | 0.32 | 0.34 | 0.18 | 0.63 | <0.01 |
| Poor appetite | Absent (Intercept) | 0.71 | 0.08 | 2.04 | 1.74 | 2.40 |  |
|  | Present | -0.41 | 0.12 | 0.66 | 0.53 | 0.83 | <0.01 |
| Weight loss | Absent (Intercept) | 0.65 | 0.08 | 1.92 | 1.65 | 2.25 |  |
|  | Present | 0.02 | 0.37 | 1.02 | 0.49 | 2.10 | 0.96 |
| Melaena | Absent (Intercept) | 0.65 | 0.08 | 1.92 | 1.65 | 2.25 |  |
|  | Present | -0.06 | 0.62 | 0.94 | 0.28 | 3.15 | 0.92 |
| Severity | Mild (Intercept) | 0.78 | 0.08 | 2.18 | 1.85 | 2.57 |  |
|  | Moderate | -0.71 | 0.11 | 0.49 | 0.40 | 0.61 | <0.01 |
| Diarrhoea pattern | Large intestinal (Intercept) | 0.58 | 0.09 | 1.79 | 1.49 | 2.15 |  |
|  | Mixed | 0.13 | 0.12 | 1.14 | 0.90 | 1.44 | 0.28 |
|  | Small intestinal | 0.22 | 0.09 | 1.24 | 1.03 | 1.50 | 0.02 |
|  | Unknown | -0.23 | 0.15 | 0.80 | 0.59 | 1.07 | 0.14 |
| Body temperature | Normal / <39 °C (Intercept) | 0.80 | 0.09 | 2.23 | 1.88 | 2.65 |  |
|  | Not recorded | -0.41 | 0.10 | 0.66 | 0.55 | 0.80 | <0.01 |
|  | 39.0 °C ≤ 39.4 °C | -0.11 | 0.12 | 0.90 | 0.71 | 1.15 | 0.40 |
|  | 39.5 °C ≤ 39.9 °C | -0.67 | 0.22 | 0.51 | 0.34 | 0.78 | <0.01 |
|  | 40.0 °C ≤ | -0.62 | 0.41 | 0.54 | 0.24 | 1.19 | 0.13 |
| **Continuous risk factor** | | | | | | | |
| Age (years) | Intercept | 0.45 | 0.10 | 1.57 | 1.30 | 1.90 |  |
|  | Age - linear | 0.02 | 0.08 | 1.02 | 0.88 | 1.18 | 0.83 |
|  | Age - quadratic | 0.30 | 0.08 | 1.35 | 1.16 | 1.56 | <0.01 |
|  | Age - cubic | -0.15 | 0.04 | 0.86 | 0.79 | 0.93 | <0.01 |

^a^ Standard error

^b^ Odds ratio

^c^ 95% Confidence interval

**Table 8**

Lost to follow-up: Parameter estimates from a series of univariable mixed effects logistic regression models, modelling outcome variable ‘loss to follow-up’ against a number of categorical and continuous risk factors.

| **Variable** | **Category** | **β** | **SE ^a^** | **OR ^b^** | **Lower CI ^c^** | **Upper CI** | ***P*** |
| --- | --- | --- | --- | --- | --- | --- | --- |
| Insurance status | Uninsured (Intercept) | -1.09 | 0.08 | 0.34 | 0.29 | 0.39 |  |
|  | Insured | -0.56 | 0.11 | 0.57 | 0.46 | 0.72 | <0.01 |
| Sex | Female (Intercept) | -1.19 | 0.09 | 0.30 | 0.26 | 0.36 |  |
|  | Male | -0.05 | 0.09 | 0.95 | 0.80 | 1.13 | 0.55 |
| Neutered status | Un-neutered (Intercept) | -0.63 | 0.09 | 0.53 | 0.44 | 0.64 |  |
|  | Neutered | -0.93 | 0.09 | 0.40 | 0.33 | 0.48 | <0.01 |
| Vaccination status | Unvaccinated (Intercept) | -0.32 | 0.10 | 0.72 | 0.59 | 0.88 |  |
|  | Vaccinated | -1.30 | 0.10 | 0.27 | 0.22 | 0.33 | <0.01 |
| Microchip status | Un-microchipped (Intercept) | -0.93 | 0.09 | 0.40 | 0.33 | 0.47 |  |
|  | Microchipped | -0.55 | 0.09 | 0.58 | 0.48 | 0.69 | <0.01 |
| Diarrhoea | Without blood (Intercept) | -1.29 | 0.09 | 0.28 | 0.23 | 0.33 |  |
|  | With blood | 0.17 | 0.09 | 1.18 | 0.99 | 1.41 | 0.07 |
| Vomit | Absent (Intercept) | -1.27 | 0.08 | 0.28 | 0.24 | 0.33 |  |
|  | Without blood | 0.12 | 0.10 | 1.12 | 0.93 | 1.35 | 0.22 |
|  | With blood | 0.40 | 0.28 | 1.49 | 0.86 | 2.60 | 0.15 |
| Other clinical signs | Absent (Intercept) | -1.23 | 0.08 | 0.29 | 0.25 | 0.34 |  |
|  | Present | 0.39 | 0.34 | 1.48 | 0.76 | 2.90 | 0.25 |
| Poor appetite | Absent (Intercept) | -1.25 | 0.08 | 0.29 | 0.25 | 0.34 |  |
|  | Present | 0.20 | 0.13 | 1.22 | 0.95 | 1.57 | 0.12 |
| Weight loss | Absent (Intercept) | -1.21 | 0.08 | 0.30 | 0.26 | 0.35 |  |
|  | Present | -0.74 | 0.50 | 0.48 | 0.18 | 1.26 | 0.14 |
| Melaena | Absent (Intercept) | -1.22 | 0.08 | 0.29 | 0.25 | 0.34 |  |
|  | Present | 0.87 | 0.62 | 2.39 | 0.71 | 8.05 | 0.16 |
| Severity | Mild (Intercept) | -1.29 | 0.08 | 0.28 | 0.24 | 0.32 |  |
|  | Moderate | 0.39 | 0.12 | 1.48 | 1.18 | 1.86 | <0.01 |
| Diarrhoea pattern | Large intestinal (Intercept) | -1.30 | 0.09 | 0.27 | 0.23 | 0.33 |  |
|  | Mixed | 0.16 | 0.13 | 1.18 | 0.91 | 1.52 | 0.21 |
|  | Small intestinal | 0.18 | 0.10 | 1.20 | 0.98 | 1.47 | 0.09 |
|  | Unknown | -0.12 | 0.18 | 0.89 | 0.63 | 1.25 | 0.49 |
| Body temperature | Normal / <39 °C (Intercept) | -1.17 | 0.09 | 0.31 | 0.26 | 0.37 |  |
|  | Not recorded | -0.11 | 0.11 | 0.90 | 0.72 | 1.11 | 0.33 |
|  | 39.0 °C ≤ 39.4 °C | -0.28 | 0.14 | 0.75 | 0.57 | 1.00 | 0.05 |
|  | 39.5 °C ≤ 39.9 °C | 0.17 | 0.23 | 1.19 | 0.75 | 1.88 | 0.46 |
|  | 40.0 °C ≤ | 0.60 | 0.42 | 1.82 | 0.81 | 4.09 | 0.15 |
| Systemic  antimicrobial | Not prescribed (Intercept) | -1.30 | 0.09 | 0.27 | 0.23 | 0.33 |  |
|  | Prescribed | 0.15 | 0.09 | 1.16 | 0.97 | 1.39 | 0.11 |
| Systemic  anti-inflammatory | Not prescribed (Intercept) | -1.24 | 0.08 | 0.29 | 0.25 | 0.34 |  |
|  | Prescribed | 0.16 | 0.14 | 1.17 | 0.89 | 1.53 | 0.25 |
| Gastrointestinal agent | Not prescribed (Intercept) | -1.33 | 0.09 | 0.26 | 0.22 | 0.31 |  |
|  | Prescribed | 0.28 | 0.09 | 1.33 | 1.11 | 1.59 | <0.01 |
| Endoparasiticide / endectocide | Not prescribed (Intercept) | -1.20 | 0.08 | 0.30 | 0.26 | 0.35 |  |
|  | Prescribed | -0.18 | 0.16 | 0.84 | 0.62 | 1.14 | 0.25 |
| Gastrointestinal nutraceutical | Not dispensed (Intercept) | -1.19 | 0.10 | 0.31 | 0.25 | 0.37 |  |
|  | Dispensed | -0.05 | 0.10 | 0.95 | 0.79 | 1.15 | 0.58 |
| Dietary modification | Not advised (Intercept) | -1.08 | 0.10 | 0.34 | 0.28 | 0.41 |  |
|  | Advised | -0.20 | 0.10 | 0.82 | 0.67 | 0.99 | 0.04 |
| Fasting | Not advised (Intercept) | -1.23 | 0.08 | 0.29 | 0.25 | 0.34 |  |
|  | Advised | 0.06 | 0.12 | 1.06 | 0.84 | 1.34 | 0.62 |
| **Continuous risk factor** | | | | | | | |
| Age (years) | Intercept | -1.13 | 0.10 | 0.32 | 0.27 | 0.39 |  |
|  | Age - linear | -0.17 | 0.08 | 0.85 | 0.72 | 1.00 | 0.05 |
|  | Age - quadratic | -0.18 | 0.08 | 0.84 | 0.71 | 0.99 | 0.03 |
|  | Age - cubic | 0.14 | 0.05 | 1.15 | 1.05 | 1.26 | <0.01 |

^a^ Standard error

^b^ Odds ratio

^c^ 95% Confidence interval

**Table 9**

Day 10 resolution: Parameter estimates from a series of univariable mixed effects logistic regression models, modelling outcome variable ‘diarrhoea resolution at 10 days post-initial presentation’ against a number of categorical and continuous risk factors.

| **Variable** | **Category** | **β** | **SE ^a^** | **OR ^b^** | **Lower CI ^c^** | **Upper CI** | ***P*** |
| --- | --- | --- | --- | --- | --- | --- | --- |
| Insurance status | Uninsured (Intercept) | 3.15 | 0.18 | 23.33 | 16.31 | 33.36 |  |
|  | Insured | -0.12 | 0.22 | 0.89 | 0.58 | 1.37 | 0.60 |
| Sex | Female (Intercept) | 3.01 | 0.19 | 20.24 | 13.84 | 29.59 |  |
|  | Male | 0.25 | 0.20 | 1.28 | 0.87 | 1.88 | 0.21 |
| Neutered status | Un-neutered (Intercept) | 3.16 | 0.24 | 23.51 | 14.66 | 37.72 |  |
|  | Neutered | -0.05 | 0.22 | 0.96 | 0.62 | 1.47 | 0.84 |
| Vaccination status | Unvaccinated (Intercept) | 2.98 | 0.25 | 19.65 | 12.09 | 31.92 |  |
|  | Vaccinated | 0.19 | 0.23 | 1.21 | 0.76 | 1.90 | 0.42 |
| Microchip status | Un-microchipped (Intercept) | 3.16 | 0.21 | 23.66 | 15.61 | 35.87 |  |
|  | Microchipped | -0.07 | 0.20 | 0.93 | 0.63 | 1.38 | 0.73 |
| Diarrhoea | Without blood (Intercept) | 3.20 | 0.19 | 24.60 | 16.86 | 35.88 |  |
|  | With blood | -0.20 | 0.20 | 0.82 | 0.56 | 1.20 | 0.31 |
| Vomit | Absent (Intercept) | 3.06 | 0.18 | 21.35 | 14.93 | 30.52 |  |
|  | Without blood | 0.28 | 0.22 | 1.32 | 0.86 | 2.05 | 0.21 |
|  | With blood | -0.99 | 0.46 | 0.37 | 0.15 | 0.92 | 0.03 |
| Other clinical signs | Absent (Intercept) | 3.12 | 0.17 | 22.54 | 16.04 | 31.67 |  |
|  | Present | 0.59 | 1.03 | 1.81 | 0.24 | 13.51 | 0.56 |
| Poor appetite | Absent (Intercept) | 3.11 | 0.18 | 22.45 | 15.85 | 31.79 |  |
|  | Present | 0.09 | 0.30 | 1.10 | 0.62 | 1.96 | 0.75 |
| Weight loss | Absent (Intercept) | 3.12 | 0.17 | 22.58 | 16.04 | 31.79 |  |
|  | Present | 0.44 | 1.03 | 1.55 | 0.21 | 11.62 | 0.67 |
| Melaena | Absent (Intercept) | 3.14 | 0.18 | 23.20 | 16.44 | 32.74 |  |
|  | Present | -1.37 | 1.13 | 0.25 | 0.03 | 2.31 | 0.22 |
| Severity | Mild (Intercept) | 3.13 | 0.18 | 22.78 | 16.01 | 32.41 |  |
|  | Moderate | -0.02 | 0.27 | 0.98 | 0.58 | 1.68 | 0.95 |
| Diarrhoea pattern | Large intestinal (Intercept) | 3.36 | 0.22 | 28.70 | 18.59 | 44.32 |  |
|  | Mixed | -0.51 | 0.28 | 0.60 | 0.35 | 1.03 | 0.06 |
|  | Small intestinal | -0.18 | 0.24 | 0.83 | 0.52 | 1.34 | 0.45 |
|  | Unknown | -0.74 | 0.31 | 0.48 | 0.26 | 0.88 | 0.02 |
| Body temperature | Normal / <39 °C (Intercept) | 3.15 | 0.14 | 23.40 | 17.84 | 30.69 |  |
|  | Not recorded | -0.20 | 0.23 | 0.82 | 0.52 | 1.29 | 0.40 |
|  | 39.0 °C ≤ 39.4 °C | -0.01 | 0.31 | 0.99 | 0.55 | 1.81 | 0.98 |
|  | 39.5 °C ≤ 39.9 °C | 1.29 | 1.06 | 3.62 | 0.45 | 28.86 | 0.23 |
|  | 40.0 °C ≤ | 15.08 | 1000< | 1000< | 0.00 | 1000< | 0.99 |
| Systemic  antimicrobial | Not prescribed (Intercept) | 3.15 | 0.20 | 23.36 | 15.76 | 34.61 |  |
|  | Prescribed | -0.06 | 0.20 | 0.95 | 0.64 | 1.39 | 0.77 |
| Systemic  anti-inflammatory | Not prescribed (Intercept) | 3.11 | 0.18 | 22.49 | 15.67 | 32.28 |  |
|  | Prescribed | -0.17 | 0.29 | 0.84 | 0.48 | 1.49 | 0.56 |
| Gastrointestinal agent | Not prescribed (Intercept) | 3.16 | 0.23 | 23.64 | 15.05 | 37.13 |  |
|  | Prescribed | -0.05 | 0.21 | 0.95 | 0.63 | 1.42 | 0.79 |
| Endoparasiticide / endectocide | Not prescribed (Intercept) | 3.19 | 0.18 | 24.18 | 16.95 | 34.49 |  |
|  | Prescribed | -0.54 | 0.28 | 0.58 | 0.34 | 1.00 | 0.05 |
| Gastrointestinal nutraceutical | Not dispensed (Intercept) | 3.16 | 0.19 | 23.62 | 16.29 | 34.24 |  |
|  | Dispensed | -0.11 | 0.20 | 0.89 | 0.60 | 1.32 | 0.58 |
| Dietary modification | Not advised (Intercept) | 3.10 | 0.23 | 22.09 | 14.11 | 34.58 |  |
|  | Advised | 0.04 | 0.22 | 1.04 | 0.68 | 1.59 | 0.85 |
| Fasting | Not advised (Intercept) | 3.15 | 0.18 | 23.27 | 16.41 | 33.00 |  |
|  | Advised | -0.18 | 0.24 | 0.84 | 0.52 | 1.35 | 0.47 |
| Dietary | Not advised (Intercept) | 3.16 | 0.18 | 23.63 | 16.69 | 33.46 |  |
| Modification alone | Advised | -0.68 | 0.42 | 0.51 | 0.22 | 1.14 | 0.10 |
| Systemic | Not prescribed (Intercept) | 0.81 | 1.02 | 22.32 | 15.85 | 31.41 |  |
| antimicrobial alone | Prescribed | 3.12 | 0.11 | 2.25 | 0.30 | 16.63 | 0.43 |
| Systemic | Not prescribed (Intercept) | 3.12 | 0.11 | 22.63 | 18.37 | 27.89 |  |
| anti-inflammatory alone | Prescribed | 13.71 | 1000< | 1000< | 0.00 | 1000< | 0.99 |
| Gastrointestinal agent | Not prescribed (Intercept) | 3.10 | 0.18 | 22.28 | 15.82 | 31.38 |  |
| alone | Prescribed | 14.95 | 73.90 | 1000< | 1000< | 1000< | 0.84 |
| Endoparasiticide / | Not prescribed (Intercept) | 3.13 | 0.18 | 22.91 | 16.26 | 32.28 |  |
| Endectocide alone | Prescribed | -1.12 | 1.10 | 0.33 | 0.04 | 2.83 | 0.31 |
| Gastrointestinal | Not dispensed (Intercept) | 3.12 | 0.18 | 22.72 | 16.10 | 32.07 |  |
| nutraceutical alone | Dispensed | 0.01 | 0.61 | 1.00 | 0.31 | 3.28 | 1.00 |
| Diet + Systemic | Not prescribed (Intercept) | 3.12 | 0.18 | 22.68 | 16.08 | 31.98 |  |
| antimicrobial alone | Prescribed | 0.04 | 0.43 | 1.04 | 0.44 | 2.43 | 0.93 |
| Diet + Systemic | Not prescribed (Intercept) | 3.09 | 0.18 | 21.97 | 15.31 | 31.53 |  |
| anti-inflammatory alone | Prescribed | -1.55 | 0.67 | 0.21 | 0.06 | 0.79 | 0.02 |
| Diet + Gastrointestinal | Not prescribed (Intercept) | 3.12 | 0.18 | 22.60 | 16.03 | 31.85 |  |
| agent alone | Prescribed | 0.16 | 0.60 | 1.17 | 0.36 | 3.82 | 0.79 |
| Diet + Endoparasiticide / | Not prescribed (Intercept) | 3.14 | 0.18 | 23.11 | 16.37 | 32.61 |  |
| Endectocide alone | Prescribed | -1.06 | 0.78 | 0.35 | 0.08 | 1.59 | 0.17 |
| Diet + Gastrointestinal | Not dispensed (Intercept) | 2.99 | 0.19 | 19.78 | 13.68 | 28.61 |  |
| nutraceutical alone | Dispensed | 0.94 | 0.40 | 2.56 | 1.18 | 5.58 | 0.02 |
| **Continuous risk factor** | | | | | | | |
| Age (years) | Intercept | 3.47 | 0.24 | 32.04 | 19.87 | 51.65 |  |
|  | Age - linear | -0.35 | 0.19 | 0.71 | 0.49 | 1.02 | 0.06 |
|  | Age - quadratic | -0.42 | 0.19 | 0.66 | 0.45 | 0.96 | 0.03 |
|  | Age - cubic | 0.20 | 0.11 | 1.22 | 0.98 | 1.53 | 0.08 |

^a^ Standard error

^b^ Odds ratio

^c^ 95% Confidence interval
